# Cross‐species chimeras reveal BamA POTRA and β‐barrel domains must be fine‐tuned for efficient OMP insertion

**DOI:** 10.1111/mmi.13052

**Published:** 2015-06-06

**Authors:** Douglas F. Browning, Vassiliy N. Bavro, Jessica L. Mason, Yanina R. Sevastsyanovich, Amanda E. Rossiter, Mark Jeeves, Timothy J. Wells, Timothy J. Knowles, Adam F. Cunningham, James W. Donald, Tracy Palmer, Michael Overduin, Ian R. Henderson

**Affiliations:** ^1^ Institute of Microbiology and InfectionUniversity of BirminghamBirminghamB15 2TTUK; ^2^ School of Cancer SciencesUniversity of BirminghamBirminghamB15 2TTUK; ^3^ College of Life SciencesUniversity of DundeeDundeeDD1 5EHUK

## Abstract

BAM is a conserved molecular machine, the central component of which is BamA. Orthologues of BamA are found in all Gram‐negative bacteria, chloroplasts and mitochondria where it is required for the folding and insertion of β‐barrel containing integral outer membrane proteins (OMPs) into the outer membrane. BamA binds unfolded β‐barrel precursors *via* the five polypeptide transport‐associated (POTRA) domains at its N‐terminus. The C‐terminus of BamA folds into a β‐barrel domain, which tethers BamA to the outer membrane and is involved in OMP insertion. BamA orthologues are found in all Gram‐negative bacteria and appear to function in a species‐specific manner. Here we investigate the nature of this species‐specificity by examining whether chimeric *E* *scherichia coli* BamA fusion proteins, carrying either the β‐barrel or POTRA domains from various BamA orthologues, can functionally replace *E* *. coli* BamA. We demonstrate that the β‐barrel domains of many BamA orthologues are functionally interchangeable. We show that defects in the orthologous POTRA domains can be rescued by compensatory mutations within the β‐barrel. These data reveal that the POTRA and barrel domains must be precisely aligned to ensure efficient OMP insertion.

## Introduction

The insertion of β‐barrel containing integral outer membrane proteins (OMPs) into the outer membrane of *Escherichia coli* is achieved by the multi‐protein β‐barrel assembly machine, the BAM complex (Knowles *et al*., 2009; Hagan *et al*., 2011). In *E. coli*, this complex molecular machine consists of the OMP BamA (BamA_Ec_: the subscript indicates the source of the BamA sequence used e.g. Ec refers to *E. coli*) and the four accessory lipoproteins (BamB–E). BamA_Ec_ is an essential protein in *E. coli* and belongs to the BamA/Omp85 family of proteins (Voulhoux *et al*., 2003; Anwari *et al*., 2012). The N‐terminal periplasmic domain of BamA_Ec_ is composed of five polypeptide transport‐associated (POTRA) motifs (POTRA_1_ to POTRA_5_) and a C‐terminal β‐barrel domain, which anchors the protein in the outer membrane (Knowles *et al*., 2009; Hagan *et al*., 2011). The POTRA domains have been shown to bind unfolded OMPs, being responsible for delivering them to the outer membrane, as well as scaffolding the BAM lipoproteins to the complex. BamD binds directly to POTRA_5_, while BamB associates through POTRA_2_ to POTRA_5_ (Kim *et al*., 2007; Knowles *et al*., 2008; Gatzeva‐Topalova *et al*., 2010; Patel and Kleinschmidt, 2013). BamC and BamE do not bind BamA_Ec_ directly, but associate with the BAM complex through BamD (Kim *et al*., 2007). The recent crystal structures of BamA_Ec_ and its orthologues have demonstrated that in BamA proteins, the C‐terminal domain folds into a 16‐stranded β‐barrel, over which the external loops (L1–L8) converge to form a covering dome (Noinaj *et al*., 2013; Albrecht *et al*., 2014; Ni *et al*., 2014). In *E. coli*, many of the loops are essential for BamA_Ec_ function, in particular L6, which is partially located within the barrel lumen because of an interaction between the barrel wall and the conserved VRGF amino acid motif at its tip (Delattre *et al*., 2010; Leonard‐Rivera and Misra, 2012; Browning *et al*., 2013; Noinaj *et al*., 2013; Rigel *et al*., 2013; Albrecht *et al*., 2014; Ni *et al*., 2014).

BAM is an evolutionary conserved machine, components of which are found in eukaryotic mitochondria and chloroplasts, as well as Gram‐negative bacteria (Voulhoux *et al*., 2003; Tommassen, 2010). BamA and BamD are essential in *E. coli* and found in all Gram‐negative bacteria. However, the other components of the complex are less conserved, for example Neisserial species lack BamB, while in some bacteria, additional accessory components are part of the BAM complex, such as Pal and BamF in *Caulobacter crescentus* and RmpM in *Neisseria meningitidis* (Volokhina *et al*., 2009; Anwari *et al*., 2010; 2012). During OMP insertion, BamA orthologues are thought to recognise the C‐terminal residues of unfolded OMPs and it has been suggested that this is species‐specific (Robert *et al*., 2006; Paramasivam *et al*., 2012). Biophysical analyses have suggested that bacterial BamA proteins function in a species‐specific manner, being optimised for folding OMPs from that species (Robert *et al*., 2006). Other studies are consistent with this as only BamA orthologues from closely related bacterial species can rescue BamA depletion in *E. coli* (Noinaj *et al*., 2013; Ruhe *et al*., 2013; Volokhina *et al*., 2013). Thus, it is surprising that *E. coli* BamA can insert mitochondrial porins into its outer membrane (Walther *et al*., 2010), while the analogous mitochondrial machinery can insert bacterial OMPs into the mitochondrial outer membrane (Walther *et al*., 2009). Therefore, to investigate this species specificity in more detail, we generated a series of chimeric BamA fusion proteins, which carry either the POTRA or barrel domains of BamA orthologues from a wide range of proteobacteria. We demonstrate that the barrel domains, and to some degree, the POTRA domains of BamA orthologues are interchangeable and that for efficient complex function, the POTRA and barrel domains must be precisely tailored.

## Results

### Rescue of BamA depletion by β‐barrel chimera fusion proteins

**Figure 1:**
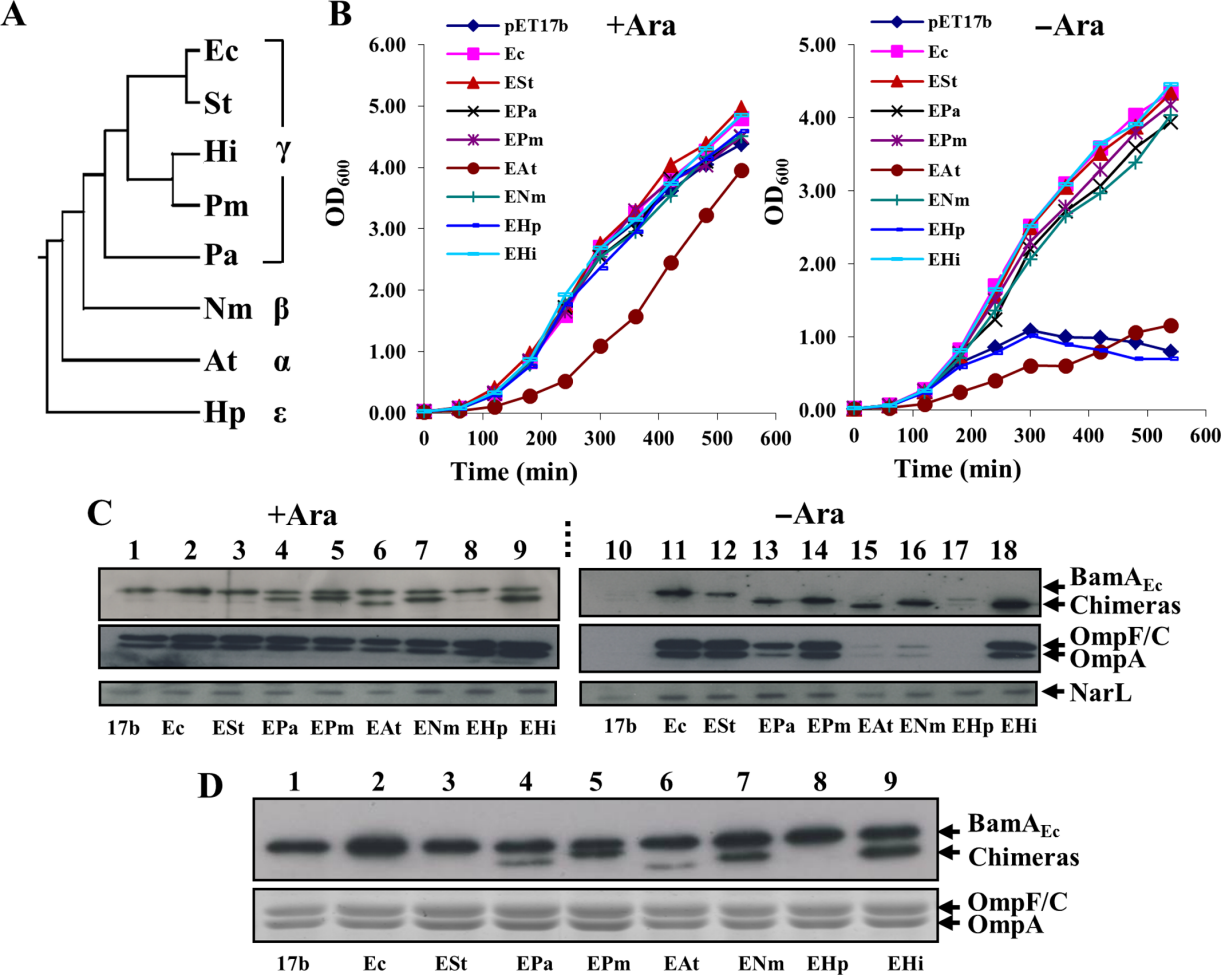
Rescue of BamA depletion by barrel chimera constructs. A. Cladistic analysis of the bacteria used in this study based on the DNA sequence of the 16S RNA genes. *E* *scherichia coli* (Ec), *S* *almonella enterica* serovar Typhimurium (St), *H* *aemophilus influenzae* (Hi), *P* *asteurella multocida* (Pm), *P* *seudomonas aeruginosa* (Pa), *N* *eisseria meningitidis* (Nm), *A* *grobacterium tumefaciens* (At) and *H* *elicobacter pylori* (Hp). B. *E* *. coli* JWD3 cells, carrying BamA barrel chimera constructs cloned into pET17b, were grown in Lennox broth supplemented with either arabinose (+ Ara) or fructose as a control (–Ara). C. Detection of BamA barrel chimeras. The panel shows Western blots of normalised total cell protein from the JWD3 cells in panel B after 300 min of growth. Blots were probed with anti‐*E* *. coli* BamA POTRA antiserum to detect BamA_Ec_ and each barrel chimera, anti‐OmpF antiserum to detect *E* *. coli* OmpF, OmpC and OmpA, and anti‐NarL antibody to detect NarL, as a loading control. D. Outer membrane containing fractions were prepared from JWD3 cells, carrying BamA barrel chimera constructs cloned into pET17b, grown in the presence of arabinose; 0.2 μg of outer membrane protein was Western blotted with anti‐*E* *. coli* BamA POTRA antiserum (top) and 2 μg was analysed using SDS‐PAGE and stained with Coomassie blue (bottom). Constructs are as follows: *E* *. coli* BamA_Ec_ (Ec), *S* *. enterica* serovar Typhimurium barrel chimera (ESt), *P* *s. aeruginosa* barrel chimera (EPa), *P* *a. multocida* barrel chimera (EPm), *A* *. tumefaciens* barrel chimera (EAt), *N* *. meningitidis* barrel chimera (ENm), *H* *e. pylori* barrel chimera (EHp) and *H* *a. influenzae* barrel chimera (EHi).

As BamA orthologues were suggested to function in a species‐specific manner (Robert *et al*., 2006; Paramasivam *et al*., 2012; Noinaj *et al*., 2013; Ruhe *et al*., 2013; Volokhina *et al*., 2013), we reasoned that species specificity would reside in the POTRA domains, and not the β‐barrels, as the POTRA domains are predicted to be the first point of contact between nascent OMPs, their periplasmic chaperones and the BAM complex. To test this hypothesis, we generated chimeric constructs in which the DNA encoding the *E. coli* BamA POTRA domains was fused in register to that encoding the barrel domains of BamA orthologues from a selection of phylogenetically diverse Gram‐negative bacteria (Fig. 1A, Table S1 and Fig. S1). As BamA_Ec_ is essential in *E. coli*, we examined whether any of the barrel chimeras rescued loss of the native BamA. The DNA encoding each fusion was cloned into vector pET17b and transformed into the previously described *E. coli* K‐12 depletion strain, JWD3 (Lehr *et al*., 2010). In JWD3 chromosomally encoded BamA_Ec_ is only produced in the presence of arabinose, while in its absence, BamA_Ec_ expression is shut down and BamA_Ec_ levels are depleted by successive cell divisions resulting in cell death; depletion can be rescued by providing a functional plasmid‐encoded copy of the *E. coli bamA*, such as pET17b/*bamA_Ec_* (Browning *et al*., 2013). Results in Fig. 1B show that, with the exception of the *Helicobacter pylori* fusion BamA_EHp_, all of the barrel chimeras supported growth in the absence of arabinose. While cells carrying the *Agrobacterium tumefaciens* BamA_EAt_ fusion grow particularly slowly, they can be repeatedly passaged in the absence of arabinose. Western blotting of total protein extracts and outer membrane preparations with anti‐*E. coli* BamA POTRA antiserum demonstrated that all the chimeras were expressed and localised to the outer membrane (Fig. 1C and D), with the exception of BamA_EHp_. Interestingly, although the *Pseudomonas aeruginosa* BamA_EPa_, *A. tumefaciens* BamA_EAt_ and *N. meningitidis* BamA_ENm_ barrel chimeras support growth in liquid culture, Western blotting using anti‐OmpF antiserum indicated that, in the absence of arabinose, the levels of OmpF, OmpC and OmpA porins were considerably lower than in cells producing BamA_Ec_ (Fig. 1C). Thus, although the barrels of most orthologues can be readily swapped to maintain viability, it is clear that some fusions function considerably better than others.

### Increased expression of β‐barrel chimeras improves function

**Figure 2:**
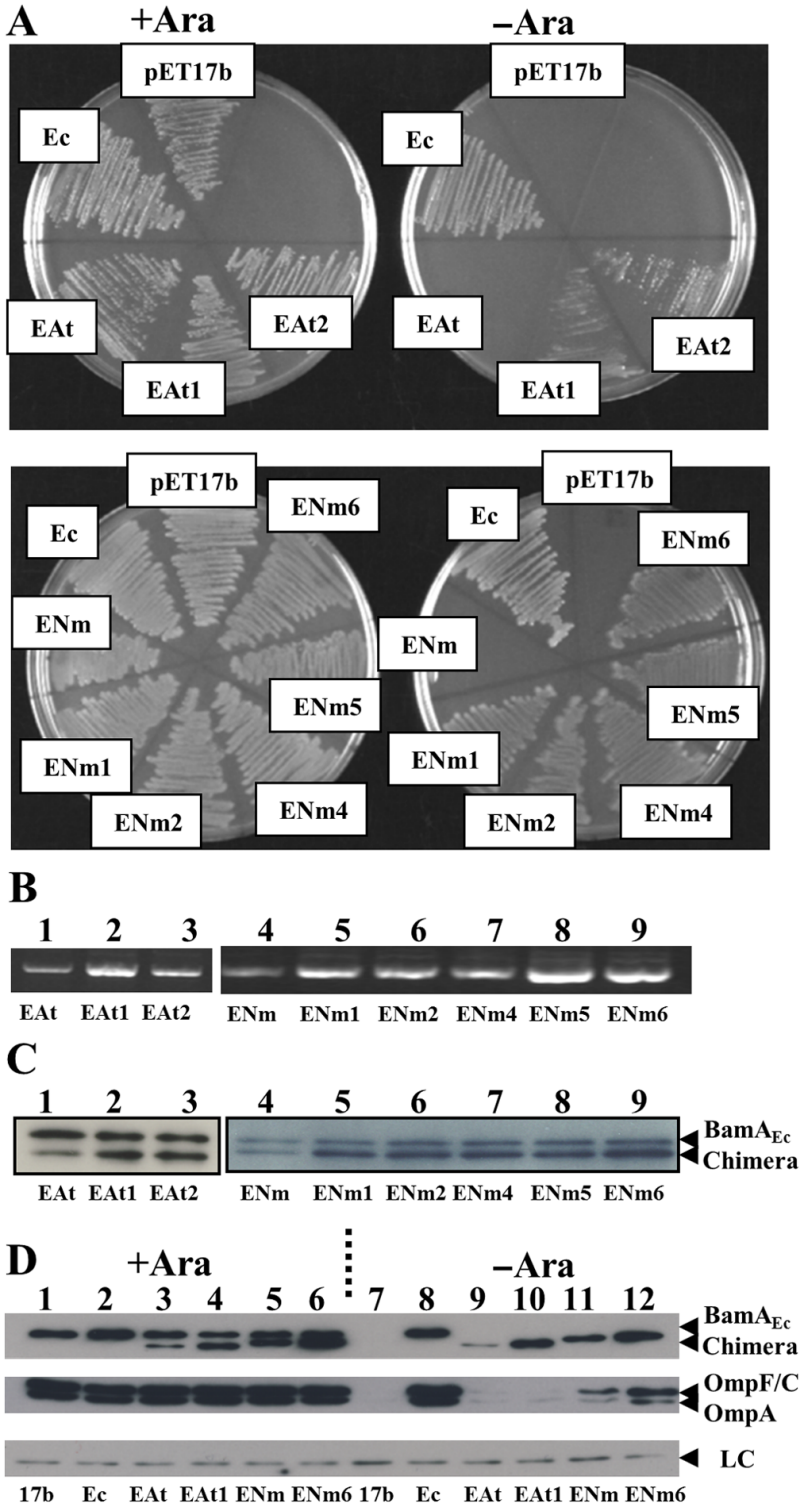
Rescue of BamA depletion by the *A* *grobacterium tumefaciens* and *N* *eisseria meningitidis* barrel chimera constructs. A. Growth of JWD3 cells on nutrient agar pates in the presence or absence of arabinose, while carrying the *A* *. tumefaciens* BamA_EAt_ (EAt) and *N* *. meningitidis* BamA_ENm_ (ENm) barrel chimeras cloned into pET17b. B. Plasmid DNA from normalised amounts of JWD3 cells, carrying different BamA_EAt_ and BamA_ENm_ constructs cloned into pET17b, was prepared using a QIAgen miniprep kit and analysed using agarose gel electrophoresis with ethidium bromide staining. C. Detection of *A* *grobacterium tumefaciens* and *N* *eisseria meningitidis* barrel chimeras. The panel shows Western blots of normalised total cell protein from the JWD3 cells carrying BamA_EAt_ and BamA_ENm_ chimeras cloned into pET17b, after 300 min of growth in Lennox broth supplemented with arabinose. Blots were probed with anti‐*E* *scherichia coli* BamA POTRA antiserum to detect BamA_Ec_, BamA_EAt_ and BamA_ENm_. D. The panel shows Western blots of normalised total cell protein from the JWD3 cells, carrying the various BamA_EAt_ and BamA_ENm_ chimeras, after 300 min of growth in Lennox broth supplemented with either arabinose (+ Ara) or fructose (–Ara). Blots were probed with anti‐*E* *. coli* BamA POTRA antiserum to detect BamA_Ec_, BamA_EAt_ and BamA_ENm_ and with anti‐OmpF antiserum to detect OmpF, OmpC and OmpA. A non‐specific band is used as a loading control (LC).

During our study, we noted that the BamA_EPa_, BamA_EAt_ and BamA_ENm_ barrel chimeras rescued depletion in liquid culture (Fig. 1B), but that JWD3 cells carrying these fusions did not form colonies on agar plates without arabinose (Fig. S2A). The reason for this is unclear, but may reflect increased stress associated with growth on solid surfaces. Therefore, to isolate chimeric constructs, which rescued depletion on solid medium, we passaged JWD3 cells, carrying pET17b/*bamA_EPa_*_,_ pET17b/*bamA_EAt_* or pET17b/*bamA_ENm_* in liquid culture and plated cells onto agar plates without arabinose. Using this strategy, we isolated plasmids encoding BamA_EPa_, BamA_EAt_ and BamA_ENm_ constructs, which rescued BamA depletion on solid medium (Fig. 2A and S3). Surprisingly, sequencing of each plasmid construct failed to detect any differences in the DNA sequence encoding each chimera, but rather point mutations were identified in the sequence encoding the RNA I/II copy number control region of pET17b (Fig. S2B). To investigate whether these mutations had an impact on plasmid copy number, we isolated plasmid DNA from similar numbers of JWD3 cells transformed with either the initial plasmid constructs encoding BamA_EPa_, BamA_EAt_ and BamA_ENm_ or their cognate evolved plasmids. Agarose gel electrophoresis revealed more plasmid DNA was isolated from strains harbouring the evolved plasmids suggesting that the copy number of each plasmid had increased (Fig. 2B and S3). Western immunoblotting of whole‐cell fractions revealed that each of the strains harbouring the evolved plasmids produced higher levels of the chimeric BamA (Fig. 2 and S3) and in liquid culture growth rates under depletion conditions resembled those of strains harbouring native BamA_Ec_ (Fig. S2C and D). Thus, the ability of these barrel chimeras to rescue depletion can be improved by increasing their cellular concentrations to that resembling native *E. coli* BamA.

### TpsB β‐barrels do not functionally substitute for BamA β‐barrels

**Figure 3:**
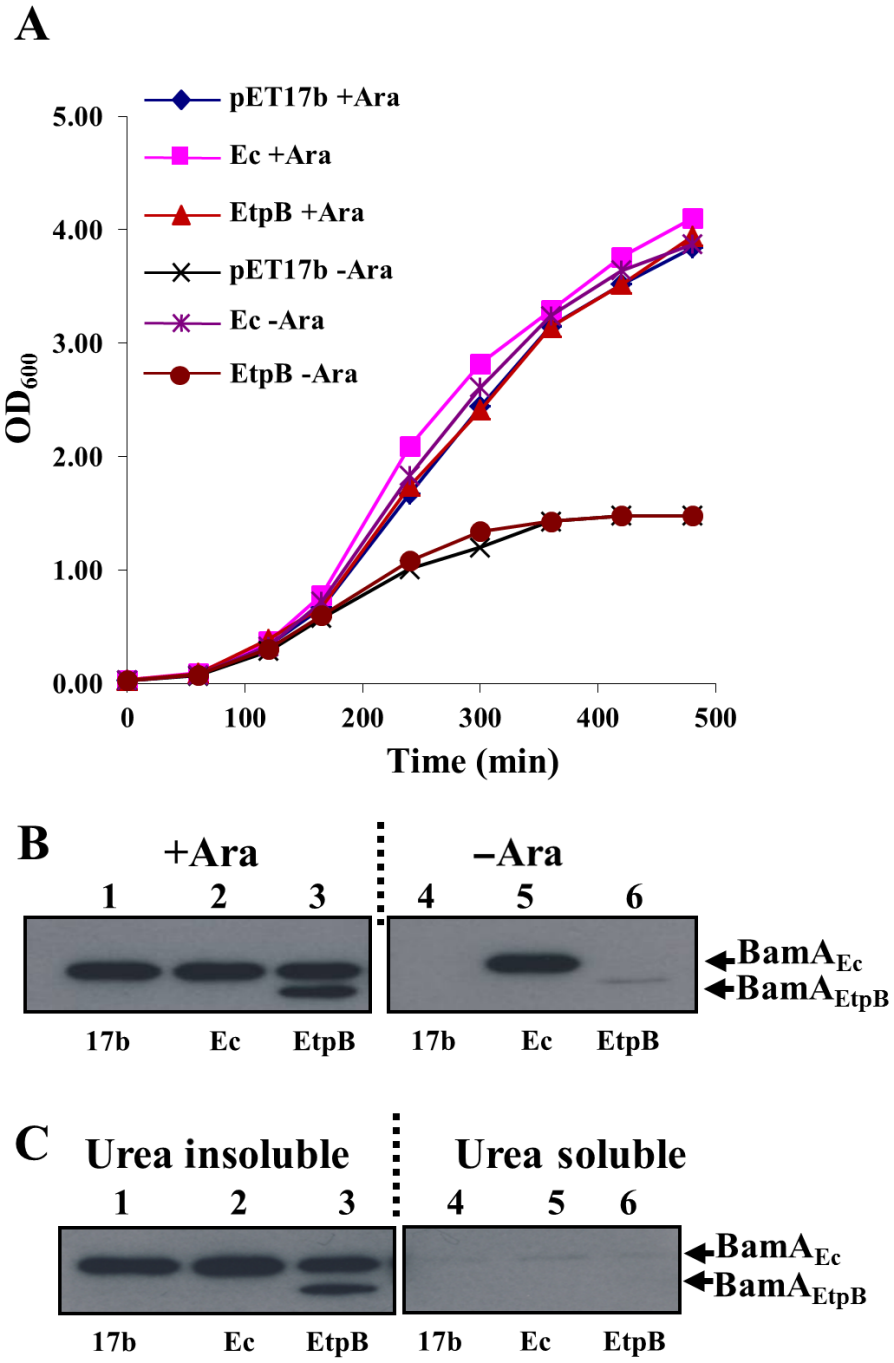
Analysis of the BamA_EtpB_ barrel chimera. A. E*scherichia coli* JWD3 cells, carrying the BamA_EtpB_ barrel chimera cloned into pET17b, were grown in Lennox broth supplemented with arabinose (+Ara) or fructose (−Ara). B. Detection of the BamA_EtpB_ barrel chimera. The panel shows Western blots of normalised total cell protein from the JWD3 cells in panel A after 300 min of growth. Blots were probed with anti‐*E* *. coli* BamA POTRA antiserum to detect BamA_Ec_ and the BamA_EtpB_ barrel chimera. C. Outer membranes preparations from JWD3 cells grown in the presence of arabinose were incubated with 5 M of urea in PBS to solubilise membrane‐associated protein aggregates. Samples were analysed using SDS‐PAGE analysis and Western blotted with anti‐*E* *. coli* BamA POTRA antiserum. Results show that both BamA_Ec_ and BamA_EtpB_ are correctly folded into the outer membrane as they localised to the urea insoluble fraction.

The BamA/Omp85 superfamily includes distantly related members such as the TpsB β‐barrels of the two‐partner secretion systems e.g. FhaC from *Bordetella pertussis* and EtpB from enterotoxigenic *E. coli* (Fleckenstein *et al*., 2006; Clantin *et al*., 2007; Meli *et al*., 2009). These TpsB proteins recognise specific proteins rich in β‐strands and secrete them across the outer membrane, whereas BamA orthologues fold β‐stranded OMPs into the membrane. Thus, we wished to investigate whether a β‐barrel domain derived from a TpsB protein would be able to functionally replace that of BamA_Ec_. To test this, we fused the DNA encoding the BamA_Ec_ POTRA domains with that encoding the EtpB barrel, to generate an EtpB barrel chimera construct (BamA_EtpB_) (Table S1 and Fig. S1). Results in Fig. 3 demonstrate that while the BamA_EtpB_ fusion was produced and folded into the outer membrane it was unable to rescue BamA depletion in JWD3, suggesting that only barrels from true BamA orthologues can functionally replace that of *E. coli* BamA.

### Rescue of BamA depletion by POTRA chimera fusion proteins

**Figure 4:**
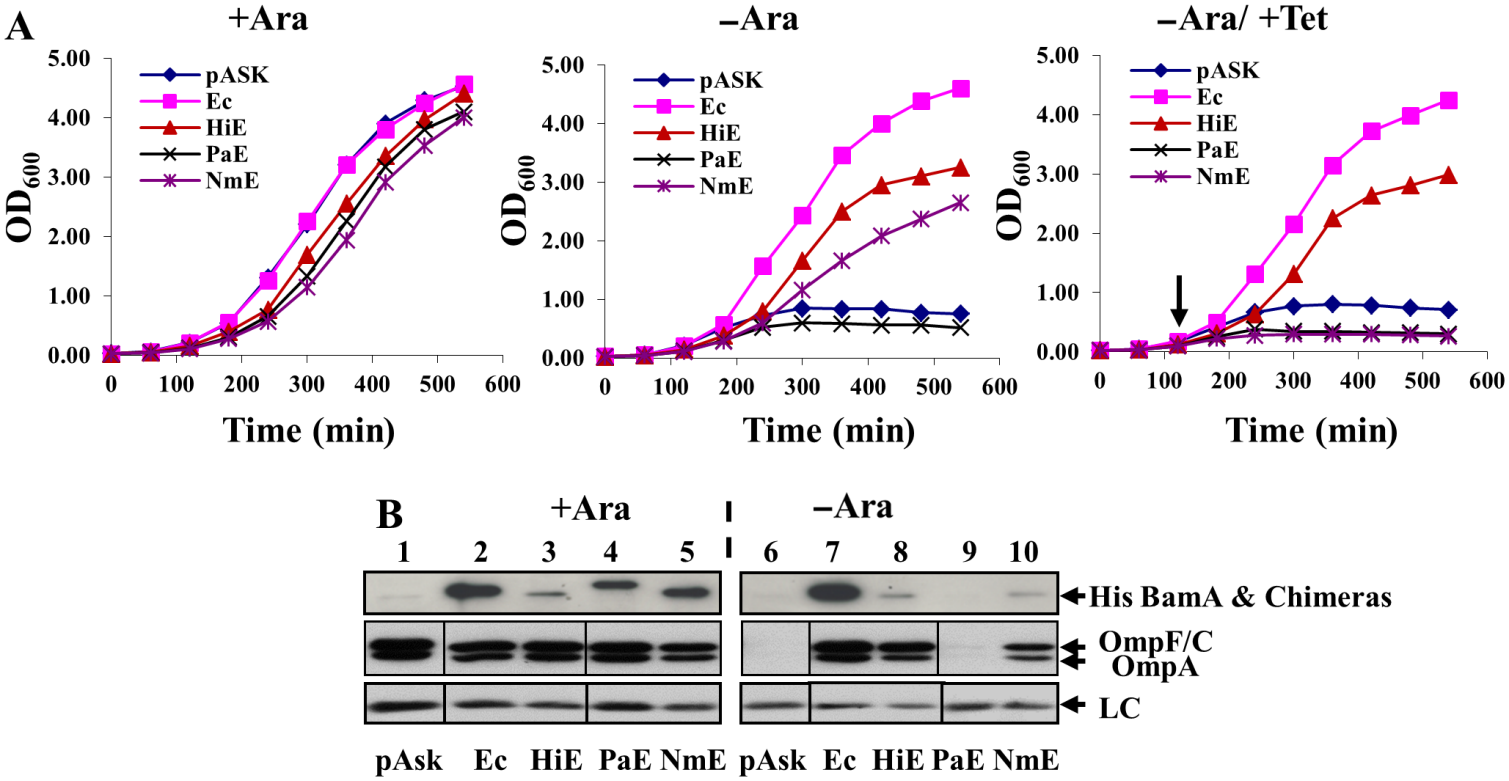
Rescue of BamA depletion by POTRA chimera constructs. A. *E* *scherichia coli* JWD3 cells, carrying BamA POTRA chimeras cloned into pASK, were grown in Lennox broth supplemented with either arabinose (+Ara), fructose (−Ara) or induced at low levels with 2 ng ml^−1^ of anhydrotetracycline (−Ara/+Tet). The point of induction is shown by an arrow. B. Detection of His‐BamA POTRA chimeras. The panel shows Western blots of normalised total cell protein from the JWD3 cells, carrying His‐BamA POTRA chimeras cloned into pASK, after 300 min of growth in Lennox broth supplemented with either arabinose (+ Ara) or fructose (−Ara). Blots were probed with anti‐His antiserum to detect His‐BamA_Ec_ and His‐BamA POTRA chimeras, anti‐OmpF antiserum to detect OmpF, OmpC and OmpA. A non‐specific band is used as a loading control (LC). Constructs are labelled as follows: *E* *. coli* BamA_Ec_ (Ec), *H* *aemophilus influenzae* POTRA chimera (HiE), *P* *seudomonas aeruginosa* POTRA chimera (PaE) and *N* *eisseria meningitidis* POTRA chimera (NmE). Note: to ensure the same sample loading order occurs throughout panel B, the order of OmpF/C/A and loading control samples has been changed with respect to the original Western blot image.

To evaluate the role of the POTRA domains in BamA species‐specificity, we generated a second series of chimeric constructs in which the POTRA domain of each orthologue was fused to the barrel of BamA_Ec_ (Table S1 and Fig. S1). Initially, gene fusions were subcloned into pET17b and their ability to rescue BamA depletion was examined in liquid medium in JWD3. Results in Fig. S4 show that only the *Salmonella enterica* serovar Typhimurium POTRA chimera (BamA_StE_) supported growth of JWD3 cells in the absence of arabinose. As the cellular level of BamA is important (Aoki *et al*., 2008), we hypothesised increased expression of the fusion proteins would allow them to rescue depletion of BamA. Thus, the chimeric genes encoding the *Haemophilus influenzae*, *P. aeruginosa* and *N. meningitidis* POTRA domains (BamA_HiE_, BamA_PaE_ and BamA_NmE_) were cloned into the high copy number (> 100 copies) expression vector, pASK‐IBA33plus (pASK). These new constructs contained an N‐terminal His‐tag to allow detection by immunoblotting; previous investigations revealed the His‐Tag did not have an impact on function (Kim *et al*., 2007; Browning *et al*., 2013). Results in Fig. 4A show that leaky uninduced expression from pASK enabled the BamA_HiE_ and BamA_NmE_ POTRA chimeras to rescue BamA depletion, while the BamA_PaE_ chimera did not. Interestingly, low‐level induction of BamA_NmE_ expression retarded growth, suggesting that elevated levels of BamA_NmE_ are toxic. Western blotting of whole‐cell lysates with anti‐OmpF antiserum demonstrated that in cells expressing His‐tagged versions of BamA_HiE_ and BamA_NmE_, porin levels were comparable with those observed for BamA_Ec_ (Fig. 4B). Furthermore, His versions of each POTRA chimera could be detected, but their levels were lower than that observed for BamA_Ec_, suggesting that these chimeric proteins may be unstable or poorly expressed (Fig. 4B). Thus, we conclude that the BamA_HiE_ and BamA_NmE_ POTRA chimeras can functionally replace *E. coli* BamA_Ec_, rescuing BamA depletion and facilitating *E. coli* OMP insertion.

### Mutations at the POTRA‐β‐barrel interface improve BamA_NmE_ function

**Figure 5:**
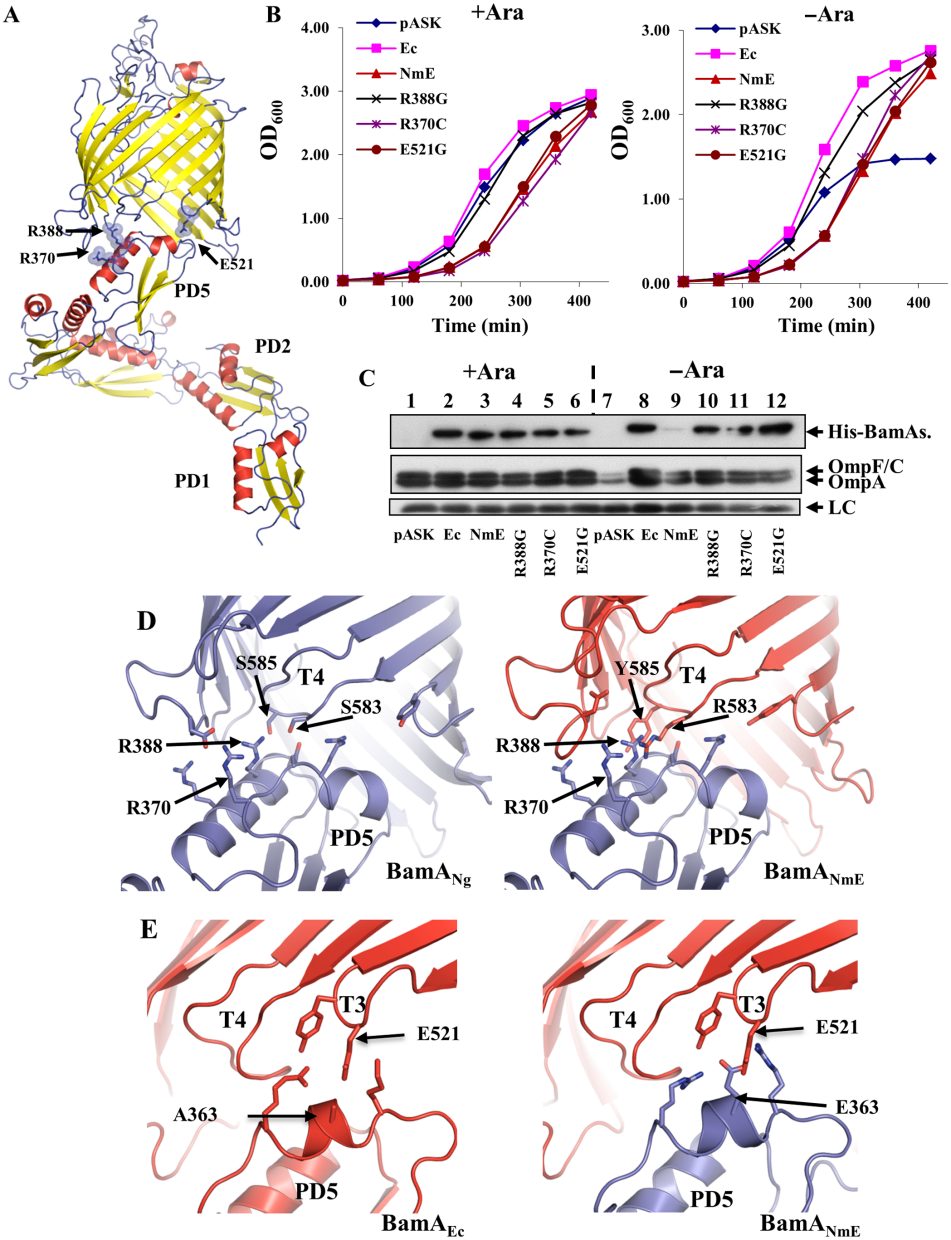
Mutational analysis of the His‐BamA_NmE_ POTRA chimera. A. The panel shows the composite structural model of the full‐length BamA_Ec_ based on the full‐length *N* *eisseria gonorrhoeae* BamA_Ng_ structure (4K3B) (Noinaj *et al*., 2013) and *E* *scherichia coli* BamA_Ec_ barrel structures (4C4V, 4N75) (Albrecht *et al*., 2014; Ni *et al*., 2014). The POTRA_4_ and POTRA_5_ structures (3OG5) have been taken from (Gatzeva‐Topalova *et al*., 2010). The positions of residues R370, R388 in POTRA_5_ and E521 in turn T3 are shown. B. *E* *. coli* JWD3 cells, carrying His‐BamA_NmE_ POTRA chimeras cloned into pASK, were grown in Lennox broth supplemented with either arabinose (+ Ara) or fructose (−Ara). C. Detection of His‐BamA_NmE_ POTRA chimeras. The panel shows Western blots of normalised total cellular protein from the JWD3 cells in panel B after 300 min of growth. Blots were probed with anti‐His tag antiserum to detect His‐BamA_Ec_ and the His‐BamA_NmE_ POTRA chimeras, and anti‐OmpF antiserum to detect OmpF, OmpC and OmpA. A non‐specific band is used as a loading control (LC). D. The panel shows the potential POTRA_5‐_barrel interactions for *N* *. gonorrhoeae* BamA_Ng_ (blue) and the BamA_NmE_ POTRA chimera (blue and red). The location of residues R370 and R388 in POTRA_5_ and S585 and S583 in turn T4 for BamA_Ng_ and the corresponding residues for BamA_NmE_ (R388, R370, Y585 and R583) are indicated. E. The panel shows the possible POTRA_5_‐barrel interactions for *E* *. coli* BamA_Ec_ (red) and the BamA_NmE_ POTRA chimera (blue and red). The location of A363 and E521 in BamA_Ec_ and E363 and E521 in BamA_NmE_, are shown. Images were prepared using PyMol (Schrodinger, 2010). Note, as some side‐chains are not present in the BamA crystal structures, their orientations have been extrapolated using most the frequent rotamers.

Although the *N. meningitidis* POTRA chimera BamA_NmE_ rescued depletion in liquid medium, it was unable to do so on agar plates (Fig. S5). To isolate versions of BamA_NmE_ that were capable of growing on agar, we repeatedly passaged JWD3 cells carrying the His‐BamA_NmE_ chimera cloned into pASK in the absence of arabinose and plated cells out onto nutrient agar plates lacking arabinose. By doing this, we isolated four candidates that rescued depletion on plates. Sequencing of the DNA encoding each chimeric construct identified each had an independent non‐synonymous substitution resulting in the following amino acid conversion: R370C, R388G, E521G and E521A (Table 1 and Fig. 5A). The R370C, R388G and E521G mutations were transferred into a clean plasmid background, and it was confirmed that only these mutations were required for this phenotype (Fig. S5). The growth of JWD3 cells, carrying these improved His‐tagged BamA_NmE_ constructs, in liquid medium was similar to the original His‐tagged BamA_NmE_ construct (Fig. 5B). However, in the absence of arabinose, the cellular levels of these proteins were considerably higher (Fig. 5C).

**Table 1:** Mutational analysis of the His‐BamA_NmE_ and BamA_Pa1–4_ POTRA chimeras

POTRA chimera.	Location of substitution.
His‐BamA_NmE_ R370C	*Neisseria meningitidis* POTRA_5_
His‐BamA_NmE_ R388G	*Neisseria meningitidis* POTRA_5_
His‐BamA_NmE_ E521G	BamA_Ec_ barrel T3
His‐BamA_NmE_ E521A	BamA_Ec_ barrel T3
BamA_Pa1–4_ T434I	BamA_Ec_ barrel L1
BamA_Pa1–4_ S436P	BamA_Ec_ barrel L1
BamA_Pa1–4_ Q441R	BamA_Ec_ barrel β2
BamA_Pa1–4_ G443D	BamA_Ec_ barrel β2
BamA_Pa1–4_ E470G	BamA_Ec_ barrel β4
BamA_Pa1–4_ D512G	BamA_Ec_ barrel β6
BamA_Pa1–4_ G528D	BamA_Ec_ barrel β7
BamA_Pa1–4_ D614G	BamA_Ec_ barrel β10
BamA_Pa1–4_ D614N	BamA_Ec_ barrel β10
BamA_Pa1–4_ A654T	BamA_Ec_ barrel L6
BamA_Pa1–4_ S657F	BamA_Ec_ barrel L6
BamA_Pa1–4_ D704N	BamA_Ec_ barrel L6
BamA_Pa1–4_ D746N	BamA_Ec_ barrel L7
BamA_Pa1–4_ N805S	BamA_Ec_ barrel β16

To understand the molecular basis for these mutations we investigated the location of each mutation in BamA using molecular models of the chimeric assemblies. The R370C and R388G mutations occurred in POTRA_5_ while the E521G and E521A mutations occurred in turn T3 of the *E. coli* barrel domain (Fig. 5D and E). In the crystal structure of *Neisseria gonorrhoeae* BamA (BamA_Ng_), POTRA_5_ makes extensive contacts with the periplasmic turns of the barrel and residues R370, R388 and E523 (equivalent to E521 from T3 in *E. coli*) are involved in this interaction network; the amino acid sequences of BamA from *N. gonorrhoeae* and from *N. meningitidis* display 96% identity (Noinaj *et al*., 2013). The available structures of BamA_Ec_ either lack POTRA domains altogether (Ni *et al*., 2014) or have POTRA_5_ pointing away from the barrel (Albrecht *et al*., 2014), making interpretation of POTRA‐barrel interactions difficult. Therefore, we modelled the POTRA_5_–barrel interactions for BamA_Ec_ and the BamA_NmE_ chimera based on the POTRA orientation observed in the BamA_Ng_ structure reported by Noinaj *et al*. (2013) and using the experimental X‐ray structures of *E. coli* barrels (Albrecht *et al*., 2014; Ni *et al*., 2014). From this comparative analysis, it is immediately obvious that steric and electrostatic clashes between R370 and R388 would arise in the chimeric BamA_NmE_ protein (Fig. 5D). In particular, R583 from T4 of the *E. coli* barrel has the capacity to clash with both R370 and R388 in BamA_NmE_. Correspondingly, the isolated R370C and R388G mutations (Fig. 5D) alleviate this clash, restoring the charge balance observed in the Neisserial protein. Furthermore, extrapolating the side‐chain orientations from the available *N. gonorrhoeae* BamA_Ng_ crystal structure, E523 (in T3) is in close proximity to E363 (POTRA_5_), which is unfavourable from electrostatic point of view. This residue pair is non‐conserved, and indeed such electrostatic repulsion is avoided in *E. coli* as position 363 in BamA_Ec_ is alanine (Fig. 5E) (Noinaj *et al*., 2013). Presuming a similar orientation of the Neisserial POTRA domains in our BamA_NmE_ POTRA chimera we expect a potential for a similar clash between E363 and E521, as in BamA_Ng_, and this is relieved by the E521G/A substitutions making this new chimeric interface more BamA_Ec_ like (Fig. 5E). Thus, we conclude that mutations in BamA_NmE_ allow POTRA_5_ to adopt different orientations with respect to the barrel domain.

### Allosteric mutations improve the function of *P* *. aeruginosa* POTRA chimera constructs

**Figure 6:**
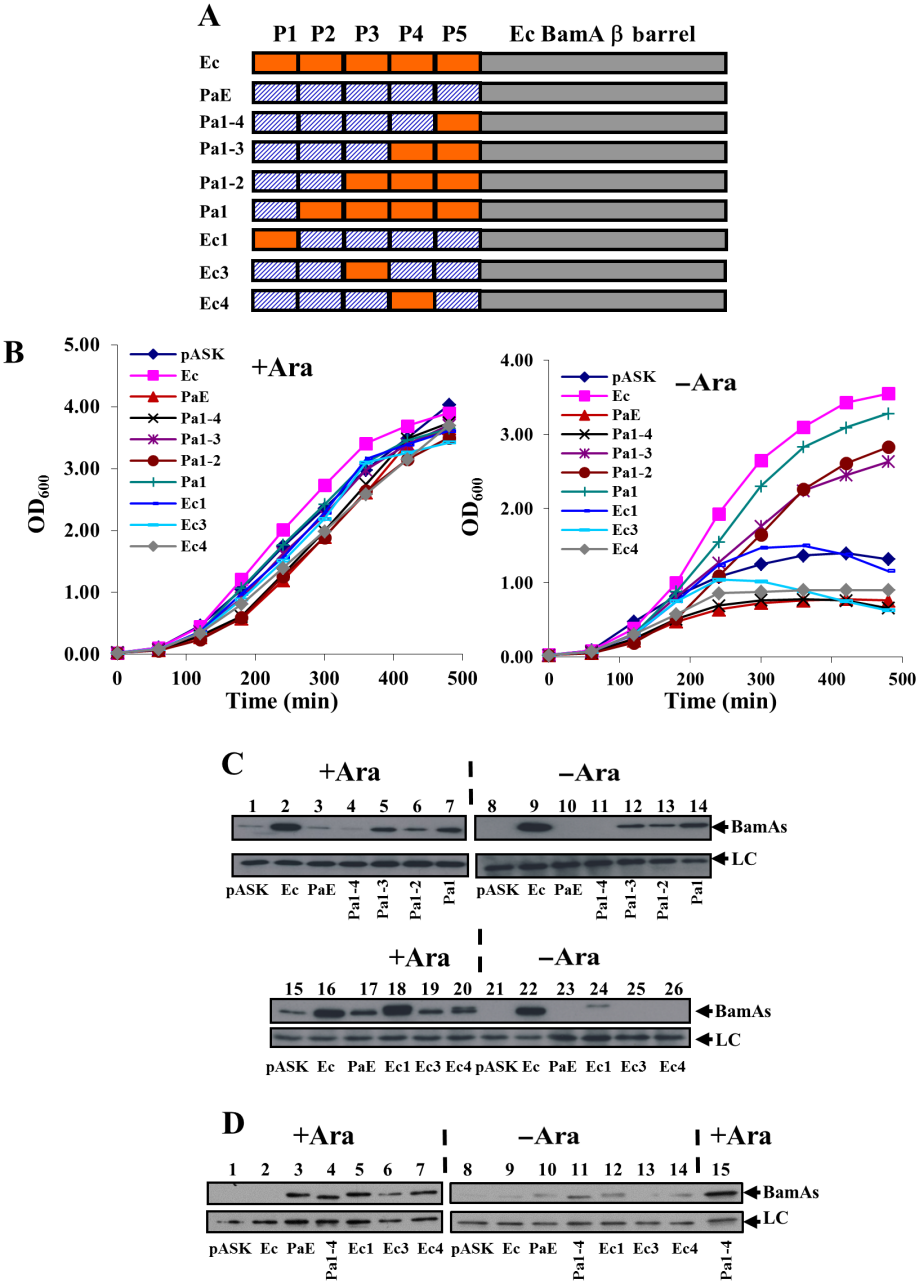
Rescue of BamA depletion by the *P* *seudomonas aeruginosa* POTRA chimeras. A. The panel shows the *P* *. aeruginosa* POTRA chimeras used in this study. The *E* *scherichia coli* POTRA domains are shown in orange, while *P* *. aeruginosa* POTRAs are shown by blue hashing. The *E* *. coli* BamA barrel is depicted as a grey rectangle. B. *E* *. coli* JWD3 cells, carrying the *P* *. aeruginosa* POTRA chimeras cloned into pASK (see panel A), were grown in Lennox broth supplemented with either arabinose (+ Ara) or fructose (−Ara). C. Detection of *P* *. aeruginosa* POTRA chimeras. The panel shows Western blots of normalised total cellular protein from JWD3 cells in panel B after 300 min of growth. Blots were probed with anti‐*E* *. coli* BamA POTRA antiserum to detect BamA_Ec_ and *P* *. aeruginosa* POTRA chimeras, where possible. A non‐specific band is used as a loading control (LC). D. Detection of *P* *. aeruginosa* POTRA chimeras with anti‐*P* *. aeruginosa* BamA antiserum. The panel shows Western blots of normalised total cellular protein from JWD3 cells in panel B after 300 min of growth. Blots were probed with anti‐*P* *. aeruginosa* BamA antiserum to detect the various *P* *. aeruginosa* POTRA chimeras. A non‐specific band is used as a loading control (LC).

The *P. aeruginosa* BamA_PaE_ POTRA chimera failed to rescue BamA depletion (Fig. 4). Initial attempts to isolate improved versions of the BamA_PaE_ chimera, by passaging plasmid constructs through the mutator strain XL‐1 Red, also failed. Therefore, to examine which *P. aeruginosa* POTRA domains prevented the chimera from functioning in *E. coli*, we generated an additional set of chimeric constructs, cloned into pASK. Starting with BamA_PaE_, we progressively replaced the *P. aeruginosa* POTRA domains with those from *E. coli* to generate constructs which possessed *P. aeruginosa* POTRA_1–4_ (BamA_Pa1–4_), POTRA_1–3_ (BamA_Pa1–3_), POTRA_1–2_ (BamA_Pa1–2_) and POTRA_1_ only (BamA_Pa1_) (Fig. 6A). We also generated constructs in which the *P. aeruginosa* POTRA_1_, POTRA_3_ and POTRA_4_ were individually replaced with those from BamA_Ec_ (i.e. BamA_Ec1_, BamA_Ec3_ and BamA_Ec4_; Fig. 6A). The ability of each construct to rescue BamA depletion was then examined in strain JWD3 in liquid culture and on agar plates. Results in Figs. 6B and S5 show that only constructs which carried both *E. coli* POTRA_4_ and POTRA_5_ could rescue BamA depletion. As these chimeric fusions carry *E. coli* POTRA domains, we examined whether fusions were detectable using Western blotting with anti‐*E. coli* POTRA antiserum. Results in Fig. 5C reveal that the BamA_Pa1–3_, BamA_Pa1–2_ and BamA_Pa1_ chimeras, which all rescue depletion, could be detected in whole‐cell preparations of BamA‐depleted cells (lanes 12–14). Western blotting using anti‐*P. aeruginosa* BamA antiserum also demonstrated that the BamA_Pa1–4_, BamA_Ec1_, BamA_Ec3_ and BamA_Ec4_ chimeric proteins were expressed in arabinose‐grown cells; however, the level of each protein was considerably lower when BamA was depleted (Fig 6D). Thus, we conclude that differences in *P. aeruginosa* POTRA_4_ and POTRA_5_ prevent the BamA_PaE_ chimera from functioning in *E. coli*.

**Figure 7:**
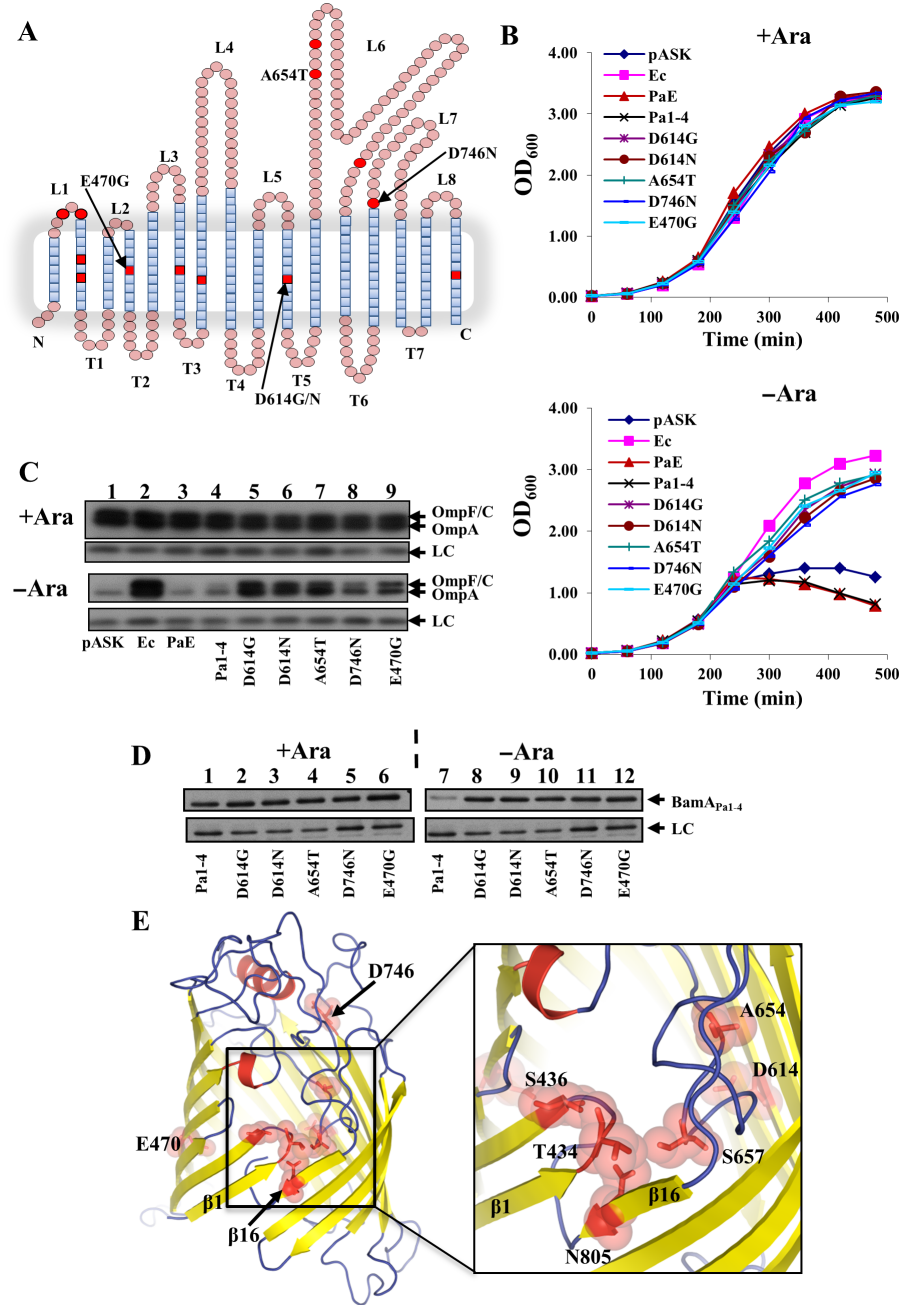
Mutational analysis of the BamA_Pa1–4_ POTRA chimera. A. A topology model of the *E* *scherichia coli* BamA β‐barrel (N422 to W810) derived from the *E* *. coli* BamA crystal structure (Albrecht *et al*., 2014). Amino acids within β‐strand regions are shown as blue squares and those in external loops and periplasmic turns are shown as pink circles. Extracellular loops L1–L8 and periplasmic turns T1–T7 are indicated. The position of mutations, which enable the BamA_Pa1–4_ POTRA chimera to rescue BamA depletion in JWD3 cells on agar plates, are red (see Table 1). The E470G, D614G, D614N, A654T and D746N substitutions were cloned into a clean plasmid background and analysed further. B. *E* *. coli* JWD3 cells, carrying BamA_Pa1–4_ POTRA chimeras cloned into pASK, were grown in Lennox broth supplemented with either arabinose (+ Ara) or fructose (−Ara). C. Detection of *E* *. coli* porins after BamA depletion. The panel shows Western blots of normalised total cell protein from the JWD3 cells, carrying the various constructs in panel B, after 300 min of growth in Lennox broth supplemented with either arabinose (+ Ara) or fructose (–Ara). Blots were probed with anti‐OmpF antiserum to detect OmpF, OmpC and OmpA, and a non‐specific band is used as a loading control (LC). D. Detection of BamA_Pa1–4_ POTRA chimeras using anti‐*P* *seudomonas aeruginosa* BamA antiserum. The panel shows Western blots of normalised total cellular protein from JWD3 cells after 300 min of growth in the presence of arabinose (+ Ara) or fructose (−Ara). Blots were probed with anti‐*P* *. aeruginosa* BamA antiserum to detect the various BamA_Pa1–4_ POTRA chimeras. A non‐specific band is used as a loading control (LC). E. The panels shows a model of the *E* *. coli* BamA β‐barrel, based on the BamA_Ec_ barrel structure (4C4V), where the missing L6 loop is replaced by L6 from the alternative BamA_Ec_ structure (4N75) and the missing C‐terminal residues are modelled in (Albrecht *et al*., 2014; Ni *et al*., 2014). A larger internal view of the barrel, focusing on the juxtaposition of L1, L6 and β16 is shown. The location of mutations, which enable the BamA_Pa1–4_ POTRA chimera to rescue BamA depletion in JWD3 cells on agar plates, is indicated (see Table 1). Images were prepared using PyMol (Schrodinger, 2010).

As the *P. aeruginosa* BamA_Pa1–4_ chimera did not rescue BamA depletion (Fig. 6), we also attempted to mutate this plasmid construct by passaging it through the XL–1 Red mutator strain. Plasmid DNA, isolated from XL‐1 Red cells, was transformed into JWD3 and cells were plated out onto on arabinose‐free agar. Using this strategy, we isolated 14 mutant constructs that rescued BamA depletion. Unexpectedly, DNA sequencing of each chimera indicated that point mutations, which enabled BamA_Pa1–4_ to function, were all located within the BamA_Ec_ β‐barrel domain and not in the POTRA domains (Table 1 and Fig. 7A). For five of these constructs (E470G, D614G, D614N, A654T and D746N), the DNA encoding each chimera was transferred into a clean plasmid background and each construct retained the ability to rescue depletion (Figs. 7B and S5). Western blotting with anti‐OmpF antiserum indicated that these improved BamA_Pa1–4_ chimeras could insert *E. coli* porins in the absence of *E. coli* BamA (Fig. 7C) and blotting with anti‐*P. aeruginosa* BamA antiserum demonstrated that in the absence of arabinose, the cellular levels of these proteins were higher than that observed for the original BamA_Pa1–4_ construct (Fig. 7D).

To understand the molecular basis for these gain‐of‐function mutations, we investigated the position of these amino acids in BamA. Many of these compensatory substitutions fall within β‐strands (e.g. E470G and D614G) and would destabilise them, increasing barrel flexibility (Merkel and Regan, 1998) (Fig. 7E). Substitution D746N disrupts the network of salt bridges, which hold loops L4, L6 and L7 together, likely making the whole barrel domain less rigid (Fig. 7E). Other mutations, such as A654T and S657F, are predicted to alter the trajectory of L6 within the barrel (Fig. 7E). We have analysed the packing of the L6 loop in the available structures, which present six non‐crystallographic copies of the loop from three different organisms. Structural superposition and B‐factor analysis (Fig. S6A) reveal striking conservation of the trajectory of the loop within the barrel in all current structures, while on the outside of the barrel it displays a high degree of flexibility, as expected for a typical non‐structured loop. The intra‐barrel section of the L6 also appears to be extremely rigid as indicated by the very low B‐factor values, and has a near identical match in all structures with a root‐mean‐square deviation from 0.22 to 0.67 Å, which is below the range of coordinate error for the structures. This extends beyond the C‐alpha atoms to the side‐chains, and is particularly evident for the VRGF motif, suggestive of the need for precise alignment of the structural elements of the loop for its functional activity (Fig. S6B). As L6 is precisely folded in all six available structures, and the L6 VGRF motif is tightly associated with the barrel (Fig. S6A), alterations of the loop will have a significant impact on the folded state of the β‐barrel, particularly S657F, which would generate a direct steric clash with β16 (Noinaj *et al*., 2013; 2014; Albrecht *et al*., 2014; Ni *et al*., 2014). In addition, mutations in L1 (T434I and S436P) and β16 (N805S) are likely to affect the pairing of β1 and β16, lowering the kinetic barrier for barrel unfolding (Fig. 7E). Thus, we conclude that defects in the POTRA domain can be bypassed by substitutions in the barrel domain, which improve the alignment of POTRA relative to the barrel and/or facilitate the opening of the barrel.

## Discussion

To understand the nature of the BAM species specificity, we generated a series of BamA β‐barrel and POTRA chimeras. Bioinformatic analyses indicated that the barrel domains of BamA orthologues are more conserved than their corresponding POTRA domains (Arnold *et al*., 2010) and consistent with this, we found that most barrel chimeras were expressed, located in the outer membrane and rescued BamA_Ec_ depletion (Fig. 1). Only the *H. pylori* barrel chimera failed to rescue BamA depletion. As we were unable to detect this protein product, this suggests that this fusion is likely unstable in *E. coli*. In contrast, Volokhina *et al*. (2013) investigated the species‐specificity observed between BamA_Ec_ and *N. meningitidis* BamA (BamA_Nm_), by expressing the full‐length BamA_Nm_ protein and similar barrel and POTRA chimeras in *E. coli*. Although protein products were detected and *E. coli* BamD was capable of binding to BamA_Nm_, all of these constructs failed to rescue depletion. In each case, gene expression was induced from plasmid pFP10 (Volokhina *et al*., 2013), while in our system, we relied on low‐level leaky expression from either pET17b or pASK. Indeed, inducing expression of the BamA_NmE_ POTRA chimera (Fig. 4A), even at low levels, retarded growth considerably, implying toxicity. Thus, we think it likely that the differences observed between the two studies are due to the different experimental systems employed.

It is of note that some barrel chimeras functioned poorly compared with the native protein. However, even slightly increasing the expression levels of these proteins greatly improved their ability to rescue depletion. As such BamA β‐barrels can functionally replace that of *E. coli*, this would suggest that the mechanism of barrel‐mediated OMP insertion has been conserved. Thus, it is quite surprising that the growth of strains expressing elevated levels of BamA_EAt_ or BamA_ENm_ were similar to the wild type on plates and in liquid medium, yet still poorly assembled porins into the outer membrane (Figs. 2 and S2).

While TpsB proteins, such as EtpB, are responsible for secreting TpsA proteins, e.g. EtpA, across the outer membrane (Fleckenstein *et al*., 2006; Meli *et al*., 2009) and BamA inserts many different OMPs into a lipid environment both recognise nascent β‐strands and have a conserved VGRF motif in L6 (Delattre *et al*., 2010). However, our BamA_EtpB_ barrel chimera failed to rescue BamA depletion in JWD3 (Fig. 3). Many of the recent crystal structures of BamA proteins demonstrate that the β1 and β16 β‐strands of BamA β‐barrels are unstably paired and it has been proposed that they separate to provide a template for OMP folding (Noinaj *et al*., 2013; 2014; Albrecht *et al*., 2014). Furthermore, it has been suggested that distortion and thinning of the lipid bilayer by the BamA β‐barrel also facilitates OMP insertion (Noinaj *et al*., 2013; Gessmann *et al*., 2014). In contrast, TpsB barrels demonstrate a high degree of barrel stability at the β1–β16 interface, and thus, it is not surprising that the BamA_EtpB_ chimera failed to rescue depletion (Clantin *et al*., 2007).

The POTRA domains act as the initial docking sites for chaperones and unfolded OMPs, as well as scaffolding the BAM lipoproteins and other associated proteins (Hagan *et al*., 2011; Webb *et al*., 2012). Therefore, we predicted that the majority of species‐specificity would reside within the POTRA domains. While it is clear that the cellular levels of some POTRA chimeras were low (Fig. 4B), suggesting that the different POTRA domains affect the biogenesis or stability of the chimeric BamA proteins, several of the POTRA chimeras folded *E. coli* porins to levels approaching that of wild‐type strains (Fig. 4B). We note that in the presence of arabinose, the levels of the POTRA chimeras are higher than in its absence (Fig. 4B). As chromosomally encoded BamA_Ec_ is expressed under these conditions in JWD3, this indicates that BamA_Ec_ is able to fold each chimeric protein more efficiently than when only each chimera is expressed. The gain‐of‐function chimeras also folded *E. coli* porins to levels approaching that of wild‐type strains. The ability of these chimeras to assemble the porins suggests that in contrast to our initial hypothesis, no species specificity resides within the POTRA domains and the accessory lipoproteins, chaperones and nascent OMPs can interact with POTRA domains from diverse species to create a functional complex.

While the BamA_PaE_ POTRA chimera failed to rescue BamA depletion because of differences in POTRA_4_ and POTRA_5_, the *P. aeruginosa* BamA_Pa1–4_ POTRA chimera, which has *P. aeruginosa* POTRA_1–4_, could be made to function by introducing substitutions within the β‐barrel (Figs. 7 and S5; Table 1). The locations of compensatory substitutions indicate that they will increase the flexibility of the BamA_Ec_ β‐barrel, facilitating more efficient communication between the POTRA and barrel domains, while others will affect the pairing of β1 and β16, influencing barrel opening by lowering the activation energy of barrel unwrapping and facilitating strand invasion by the nascent OMP chain. Indeed, in one BamA_Ec_ structure, N805 stabilises the β16 kink observed in the terminal β‐strand (Noinaj *et al*., 2013; Albrecht *et al*., 2014). It is also of note that, L1 forms part of a pore by which the external loops of folding OMPs may exit BamA and so our substitutions could also affect this process (Noinaj *et al*., 2013; 2014; Albrecht *et al*., 2014; Ni *et al*., 2014).

In conclusion, our results demonstrate that the level of BamA expression is critical for the BAM to function, and that defects in the POTRA domains can be compensated for by substitutions within the barrel. The latter data suggest that the POTRA and barrel domains communicate during OMP biogenesis and that their interactions must be fine‐tuned for efficient OMP folding. Importantly, our data reveal there is no strong amino acid template within BamA that confers species specificity for particular OMPs.

## Experimental procedures

### Bacterial strains, growth conditions, plasmids and primers

The bacterial strains, plasmids, DNA fragments and primers used in this study are detailed in Table S1. RLG221 was used as a standard *E. coli* K‐12 strain throughout and all bacteria were cultured in Lennox broth [2% (w/v) peptone (Merck, Kenilworth, NJ, USA), 1% (w/v) yeast extract (Fisher Scientific, Loughborough, UK) and 170 mM NaCl] (Squire *et al*., 2010) and on nutrient agar (Oxoid, Basingstoke, Hampshire, UK). Ampicillin (100 μg ml^−1^) was included in media where appropriate. Low‐level protein expression was induced in cells carrying pASK plasmid derivatives by the addition of anhydrotetracycline to 2 ng ml^−1^.

To determine the ability of plasmid constructs to rescue BamA depletion on solid media, the *E. coli* BamA depletion strain JWD3 was grown on agar plates in the presence or absence of 0.2% (w/v) arabinose (Lehr *et al*., 2010). To assess this in liquid media, JWD3 cells were grown in 50 ml of Lennox broth at 37 °C with shaking in the presence of 0.05% (w/v) arabinose or 0.05% (w/v) fructose, as a control, and optical density (OD_600_) was monitored over time. After 300 min growth, cultures were sampled for analysis. All growth curves were done at least twice and representative curves are shown in figures. If constructs failed to rescue depletion in the presence of fructose, no further growth was detected after this point.

### Plasmid construction

The DNA encoding each BamA orthologue was synthesised by Genscript (http://www.genscript.com) and cloned into pET17b using *Nde*I and *Xho*I. Each orthologue construct possessed the DNA encoding for the *E. coli* BamA signal sequence, ensuring efficient transit across the inner membrane, and were codon optimised for high‐level expression in *E. coli* (Fig. S1 and Table S1). To aid gene manipulation, each ORF was purged of restriction sites and unique sites for *Nde*I, *Nhe*I, *Bam*HI and *Xho*I were introduced to facilitate easy swapping of the POTRA and barrel domains (Fig. S1). POTRA and barrel chimeras were, therefore, generated by sub‐cloning the relevant *Nde*I‐*Bam*HI and *Bam*HI‐*Xho*I DNA fragments into pET17b/*bamA_Ec_*. The POTRA and barrel chimeras are designated BamA_XE_ and BamA_EX_, respectively, were X denotes the initials of the bacterial species from which the POTRA or barrel domains derives. pASK derivatives, carrying the DNA encoding BamA POTRA chimeras, were generated using PCR. DNA was amplified using the relevant pET17b construct as template, with primers BamABsaI and PetTerm. PCR products were cloned into pASK using *Bsa*I and *Xho*I. N‐terminal His epitope tags were introduced into BamA POTRA chimera constructs using PCR. DNA was amplified using the required His primer (Table S1) and BamA1372Rev, with the relevant pASK chimeric construct as template. Product was restricted with *Nhe*I and *Bam*HI and cloned into pASK/*bamA_Ec_*, placing the His tag directly after the signal sequence in each case. All DNA constructs were verified by DNA sequencing.

The BamA_EtpB_ barrel chimera construct was generated using PCR. The DNA encoding the EtpB barrel was amplified using primers EtpBUp and EtpBDown, with plasmid pJMF1002 as template (Fleckenstein *et al*., 2006). Product was restricted with *Bam*HI and *Xho*I, cloned into pET17b/*bamA_Ec_* and verified by DNA sequencing.

The swapping of individual POTRA motifs between BamA_Ec_ and BamA_PaE_ was achieved using mega‐primer PCR (Sarkar and Sommer, 1990; Rossiter *et al*., 2011a). For POTRA chimeras BamA_Pa1–4_, BamA_Pa1–3_, BamA_Pa1–2_ and BamA_Pa1_ the first‐round PCR product was generated using primer BamA1372Rev and primers Pa4Ec5, Pa3Ec4, Pa2Ec3 and Pa1Ec2 with pET17b/*bamA_Ec_* as template. PCR products were used in a second round of PCR with primer PetPro and the POTRA chimera construct pET17b/*bamA_PaE_* as template. For POTRA chimera BamA_Ec1_ the first‐round PCR used primers Ec1Pa2 and BamA1372Rev with pET17b/*bamA_PaE_* as template and primer PetPro and pET17b/*bamA_Ec_* in the second‐round PCR. Chimeras BamA_Ec3_ and BamA_Ec4_ were generated using primers BamA1372Rev and either Ec3Pa4 and Ec4Pa5 with pET17b/*bamA_PaE_* as template. In the second round, PCR products were used with primer PetPro and pASK/*bamA_Pa1–2_* and pASK/*bamA_Pa1–3_*, respectively, as template. All PCR products were cloned into pASK/*bamA_EC_* using *Nhe*I and *BamH*I and verified by DNA sequencing.

### Sample preparation and Western blotting

JWD3 cells, carrying various pET17b and pASK constructs were grown in 50 ml of Lennox broth at 37°C with shaking for 300 min in the presence of 0.05% (w/v) arabinose or fructose. The preparation of normalised total cellular protein samples, isolation of membrane fractions from cultures and the washing of membranes with urea were carried out as detailed Browning *et al*. (2013). Protein samples were resolved by SDS‐PAGE and analysed using Western blotting as in (Rossiter *et al*., 2011b). *E. coli* BamA protein was detected using anti‐*E. coli* POTRA BamA antiserum (Rossiter *et al*., 2011b), *P. aeruginosa* BamA using anti‐BamA_Pa_ antiserum, OmpF, OmpC and OmpA proteins were detected using anti‐OmpF antiserum and NarL using anti‐NarL antibodies, all raised in rabbit. N‐terminal His tags were detected using anti‐His tag mouse monoclonal antibodies (Sigma‐Aldrich, Gillingham, Dorset, UK). Blots were developed using the ECL Western Blotting Detection System (GE Healthcare, Little Chalfont, Buckinghamshire, UK).

### Structural modelling of BamA

To generate the full‐length composite model of the *E. coli* BamA_Ec_ protein (GenBank AAC73288), we used the X‐ray barrel structure of BamA_Ec_ (4C4V) (Albrecht *et al*., 2014), splicing in the missing L6 loop from the alternative Bam_Ec_ structure (4N75) (Ni *et al*., 2014) and manually building the missing C‐terminal residues using Coot (Emsley *et al*., 2010). I‐TASSER (Roy *et al*., 2010) was used with specific templates (3OG5) (Gatzeva‐Topalova *et al*., 2010) to generate an additional model for POTRA_1–5_, arranged according to the full‐length *N. gonorrhoeae* BamA_Ng_ POTRA orientation (4K3B) (Noinaj *et al*., 2014). The final models were manually optimised using Coot (Emsley *et al*., 2010). Structural superposition has been performed using Gesamt and SSM as implemented in CCP4 suite (Winn *et al*., 2011). Structural visualisations were done with PyMOL (Schrodinger, 2010).

## Conflict of interest

The authors declare no conflict of interest.

## Supporting information

Supporting information
