# Supplementary material for: Cross‐species chimeras reveal BamA POTRA and β‐barrel domains must be fine‐tuned for efficient OMP insertion
Source: Mol Microbiol. 2015 Jun 6;97(4):646–59. doi: 10.1111/mmi.13052 (PMC4950039; doi:10.1111/mmi.13052)
Supplement: Supplementary file 1 — Supporting information [file MMI-97-646-s001.pdf]

For Molecular Microbiology

Supplementary Information

Cross-species chimeras reveal BamA POTRA and β-barrel domains must be fine-tuned for efficient OMP insertion.

Douglas F. Browning[1], Vassiliy N. Bavro[1], Jessica L. Mason[1], Yanina R. Sevastsyanovich[1], Amanda E. Rossiter[1], Mark Jeeves[2], Timothy J. Wells[1], Timothy J. Knowles[2], Adam F. Cunningham[1], James W. Donald[3], Tracy Palmer[3], Michael Overduin[2] and Ian R. Henderson[1].

[1] Institute of Microbiology and Infection, University of Birmingham, Birmingham B15 2TT, UK.

[2] School of Cancer Sciences, University of Birmingham, Birmingham B15 2TT, UK.

[3] College of Life Sciences, University of Dundee, Dundee DD1 5EH, UK.

Table S1. Strains, plasmids, DNA fragments and Primers used in this work.

**Strains.**	**Relevant genotype or sequence.**	**Reference.**
RLG221	*E. coli* K-12 cloning strain. *recA56 araD139(are-leu)7697 laxX74 glaU galK hsdR strA.*	R. Gourse
JWD3	*E. coli* K-12 BamA depletion strain.	(Lehr *et al.*, 2010)
XL-1 Red	*E. coli K-12 mutator strain. endA1 gyrA96 thi-1 hsdR17 supE44 relA1 lac mutD5 mutS mutT* Tn*10* (Tet^R)	Agilent Technologies
Plasmids.		
pET17b	T7 expression vector (Amp^R).	Novagen
pASK	pASK-IBA33plus protein over-expression vector (Amp^R).	IBA
pJMF1002	EtpB-Flag expression plasmid (Amp^R).	(Fleckenstein *et al.*, 2006)
**DNA Fragments.**		
$bamA_{Ec}$	DNA fragment encoding a codon-optimised version of *E. coli* BamA.	(Browning *et al.*, 2013)
$hisbamA_{Ec}$	DNA fragment encoding an N-terminal His tagged version of the of *E. coli* BamA	(Browning *et al.*, 2013)
$bamA_{St}$	DNA fragment encoding a codon-optimised version of *S. enterica* serovar Typhimurium BamA.	This work
$bamA_{Hi}$	DNA fragment encoding a codon-optimised version of *H. influenzae* BamA (previously D15).	This work
$bamA_{Pm}$	DNA fragment encoding a codon-optimised version of *P. multocida* BamA (previously Oma87).	This work
$bamA_{Pa}$	DNA fragment encoding a codon-optimised version of *P. aeruginosa* BamA (previously Opr86).	This work
$bamA_{At}$	DNA fragment encoding a codon-optimised version of *A. tumefaciens* BamA.	This work
$bamA_{Nm}$	DNA fragment encoding a codon-optimised version of *N. meningitidis* BamA (previously Omp85).	This work
$bamA_{Hp}$	DNA fragment encoding a codon-optimised version of *H. pylori* BamA (previously D15).	This work
$bamA_{ESt}$	DNA fragment encoding the *S. enterica* Typhimurium barrel chimera fusion protein ($BamA_{ESt}$).	This work
$bamA_{EHi}$	DNA fragment encoding the *H. influenzae* barrel chimera fusion protein ($BamA_{EHi}$).	This work
$bamA_{EPm}$	DNA fragment encoding the *P. multocida* barrel chimera fusion protein ($BamA_{EPm}$).	This work
$bamA_{EPa}$	DNA fragment encoding the *P. aeruginosa* barrel chimera fusion protein ($BamA_{EPa}$).	This work
$bamA_{EAt}$	DNA fragment encoding the *A. tumefaciens* barrel chimera fusion	This work

	protein (BamA$_{EAt}$).	
bamA$_{ENm}$	DNA fragment encoding the *N. meningitidis* barrel chimera fusion protein (BamA$_{ENm}$).	This work
bamA$_{EHp}$	DNA fragment encoding the *H. pylori* barrel chimera fusion protein (BamA$_{EHp}$).	This work
bamA$_{EtpB}$	DNA fragment encoding the EtpB barrel chimera fusion protein (BamA$_{EtpB}$).	This work
bamA$_{StE}$	DNA fragment encoding the *S. enterica* Typhimurium POTRA chimera fusion protein (BamA$_{StE}$).	This work
bamA$_{HiE}$	DNA fragment encoding the *H. influenzae* POTRA chimera fusion protein (BamA$_{HiE}$).	This work
bamA$_{PmE}$	DNA fragment encoding the *P. multocida* POTRA chimera fusion protein (BamA$_{PmE}$).	This work
bamA$_{PaE}$	DNA fragment encoding the *P. aeruginosa* POTRA chimera fusion protein (BamA$_{PaE}$).	This work
bamA$_{AtE}$	DNA fragment encoding the *A. tumefaciens* POTRA chimera fusion protein (BamA$_{AtE}$).	This work
bamA$_{NmE}$	DNA fragment encoding the *N. meningitidis* POTRA chimera fusion protein (BamA$_{NmE}$).	This work
bamA$_{HpE}$	DNA fragment encoding the *H. pylori* POTRA chimera fusion protein (BamA$_{HpE}$).	This work
bamA$_{Pa1-4}$	DNA fragment encoding a POTRA chimera fusion protein carrying *P. aeruginosa* POTRA$_{1-4}$ and *E. coli* POTRA$_{5}$ (BamA$_{Pa1-4}$).	This work
bamA$_{Pa1-3}$	DNA fragment encoding a POTRA chimera fusion protein carrying *P. aeruginosa* POTRA$_{1-3}$ and *E. coli* POTRA$_{4-5}$ (BamA$_{Pa1-3}$).	This work
bamA$_{Pa1-2}$	DNA fragment encoding a POTRA chimera fusion protein carrying *P. aeruginosa* POTRA$_{1-2}$ and *E. coli* POTRA$_{3-5}$ (BamA$_{Pa1-2}$).	This work
bamA$_{Pa1}$	DNA fragment encoding a POTRA chimera fusion protein carrying *P. aeruginosa* POTRA$_{1}$ and *E. coli* POTRA$_{2-5}$ (BamA$_{Pa1}$).	This work
bamA$_{Ec1}$	DNA fragment encoding a POTRA chimera fusion protein carrying *E. coli* POTRA$_{1}$ and *P. aeruginosa* POTRA$_{2-5}$ (BamA$_{Ec1}$).	This work
bamA$_{Ec3}$	DNA fragment encoding a POTRA chimera fusion protein carrying *P. aeruginosa* POTRA$_{1-2}$ and POTRA$_{4-5}$ and *E. coli* POTRA$_{3}$ (BamA$_{Ec3}$).	This work
bamA$_{Ec4}$	DNA fragment encoding a POTRA chimera fusion protein carrying *P. aeruginosa* POTRA$_{1-3}$ and POTRA$_{5}$ and *E. coli* POTRA$_{4}$ (BamA$_{Ec4}$).	This work
hisbamA$_{HiE}$	DNA fragment encoding an N-terminal His tagged version of the *H. influenza* POTRA chimera fusion protein (His-BamA$_{HiE}$).	This work
hisbamA$_{PaE}$	DNA fragment encoding an N-terminal His tagged version of the *P. aeruginosa* POTRA chimera fusion protein (His-BamA$_{PaE}$).	This work
hisbamA$_{NmE}$	DNA fragment encoding an N-terminal His tagged version of the *N. meningitides* POTRA chimera fusion protein (His-BamA$_{NmE}$).	This work

Primers (5' to 3').

PetPro	TAATACGACTCACTATAGGG
PetTerm	GTGGCAGCAGCCAACTCAGC
pASK Fw	GAGTTATTTTACCACTCCCT
pASK Rev	CGCAGTAGCGGTAAACG
BamABsaI	GGGGGGGTCTCGAATGGCGATGAAAAAACTGCTGATCG
BamA1130F	GAAATGCGTCAGATGGAAGGTGC
BamA1372R	TCGTTTTTGGTCCCGTTGATACCAAC
HisBamA	GGGGGGCTAGCCACCACCACCACCACCACGCGGCGGAAG GTTTCGTTGTTAAAGACATC
HisBamAHi	GGGGGGCTAGCCACCACCACCACCACCACGCGGCGCCGT TCGTTGCGAAAGACATCCGTGTTG
HisBamAPa	GGGGGGCTAGCCACCACCACCACCACCACGCGGCGGAAT CTTTCACCGTTTCTGACATCCG
HisBamANm	GGGGGGCTAGCCACCACCACCACCACCACGCGGCGGACT TCACCATCCAGGACATCCGTGTTG
Pa1Ec2	GTTGTTACCGTTGTTGAACGTCCGACCATCGCGTCTATCA CC
Pa2Ec3	CAACATCAACGAAGGCACCGTTGCGGAAATCCAGCAGAT CAAC
Pa3Ec4	GAACATCAACGAAGGTGAAAAATACAAACTGTCTGGTGT TGAAG
Pa4Ec5	CGTTGTTGACCCAGGTAAACGTTTCTACGTTCGTAAAATC CG
Ec1Pa2	CTGGTTCAGGTTAAAGAACGTCCGTCTATCTCTTCTATCG AAATCG
Ec3Pa4	CCGTGAACATCACCGAAGGTGACCAGTACACCATCCGTG ACGTTAAACTG
Ec4Pa5	GTGAACGTGGACGCGGGTAACCGTGCGTATGTGAACCGT ATCAACTTCC
EtpBUp	GGGGGATCCCGTGCCGGCGGGTATGTGGGGC
EtpBDown	GGGGCTCGAGTCAGAACGTTTTCAGGGCTGAC

Supporting Information Figure Legends.

Fig. S1. DNA and protein sequences of BamA orthologues used in this study.

A. The DNA sequences of codon optimized *bamA* constructs used in this study. Restriction enzyme recognition sites, located within each *bamA* orthologue, were removed and *Nde*I, *Nhe*I, *Bam*HI and *Xho*I were introduced at the positions indicated (shown as bold and underlined) to facilitate gene manipulation of the DNA encoding the POTRA and barrel domains. The fragments shown are from *E. coli* ($bamA_{Ec}$), *S. enterica* serovar Typhimurium ($bamA_{St}$), *P. aeruginosa* ($bamA_{Pa}$), *P. multocida* ($bamA_{Pm}$), *A. tumefaciens* ($bamA_{At}$), *N. meningitidis* ($bamA_{Nm}$), *H. pylori* ($bamA_{Hp}$), *H. influenzae* ($bamA_{Hi}$), and the EtpB barrel chimera fusion ($bamA_{EtpB}$).

B. Amino acid sequences of the full length BamA orthologues used in this study. Note that each BamA orthologue possesses the N-terminal signal sequence from wild-type *E. coli* BamA protein (underlined) to ensure that each protein was correctly targeted to the *E. coli* SEC translocon. The protein sequences displayed are from *E. coli* ($\mathrm{BamA_{Ec}}$), *S. enterica* serovar Typhimurium ($\mathrm{BamA_{St}}$), *P. aeruginosa* ($\mathrm{BamA_{Pa}}$) *P. multocida* ($\mathrm{BamA_{Pm}}$), *A. tumefaciens* ($\mathrm{BamA_{At}}$), *N. meningitidis* ($\mathrm{BamA_{Nm}}$), *H. pylori* ($\mathrm{BamA_{Hp}}$), *H. influenzae* ($\mathrm{BamA_{Hi}}$), and the EtpB barrel chimera fusion ($\mathrm{BamA_{EtpB}}$).

Fig. S2. Rescue of BamA depletion by BamA barrel chimeras.

A. Growth of JWD3 cells on nutrient agar pates in the presence or absence of arabinose, whilst carrying BamA barrel chimeras cloned into pET17b. Constructs were as follows: *E. coli* $\mathrm{BamA_{Ec}}$ (Ec), *S. enterica* serovar Typhimurium barrel chimera (ESt), *P. aeruginosa* barrel chimera (EPa), *P. multocida* barrel chimera (EPm), *A. tumefaciens* barrel chimera (EAt), *N. meningitidis* barrel chimera (ENm), *H. pylori* barrel chimera (EHp) and *H. influenzae* barrel chimera (EHi).

B. The panel shows the DNA sequence of the pET17b copy number control region and the location of mutations, which allow the *A. tumefaciens* $\mathrm{BamA_{EAt}}$ barrel chimera constructs (EAt1 and EAt2), the *N. meningitidis* $\mathrm{BamA_{ENm}}$ barrel chimera constructs (ENm1, ENm2, ENm4, ENm5 and ENm6) and *P. aeruginosa* barrel chimera constructs (EPa1 and EPa2) to rescue BamA depletion on agar plates. The -35 and -10 promoter elements and the transcription start sites of RNA I and RNA II are shown (Camps, 2010).

C. *E. coli* JWD3 cells, carrying different $\mathrm{BamA_{EAt}}$ constructs cloned into pET17b, were grown in Lennox broth supplemented with arabinose (+Ara) or fructose (-Ara).

D. *E. coli* JWD3 cells, carrying BamA_{ENm} barrel chimeras constructs cloned into pET17b, were grown in Lennox broth supplemented with arabinose (+Ara) or fructose (-Ara).

Fig. S3. Rescue of BamA depletion by the *P. aeruginosa* barrel chimera constructs.

A. Growth of JWD3 cells on nutrient agar pates in the presence or absence of arabinose, whilst carrying various *P. aeruginosa* BamA_{EPa} (EPa) barrel chimera constructs cloned into pET17b.

B. Plasmid DNA from normalised amounts of JWD3 cells, carrying different BamA_{EPa} constructs cloned into pET17b, was prepared using a QIAgen miniprep kit and analysed using agarose gel electrophoresis with ethidium bromide staining.

C. Detection of *P. aeruginosa* barrel chimeras. The panel shows Western blots of normalised total cell protein from JWD3 cells carrying BamA_{EPa} chimeras cloned into pET17b, after 300 minutes of growth in Lennox broth supplemented with arabinose. Blots were probed with anti-*E. coli* BamA POTRA antiserum to detect BamA_{Ec} and BamA_{EPa}.

Fig. S4. Rescue of BamA depletion by POTRA chimera constructs.

E. coli JWD3 cells, carrying BamA POTRA chimeras cloned into pET17b, were grown in Lennox broth supplemented with arabinose (+Ara) or fructose (-Ara). Constructs are as follows: *E. coli* BamA_{Ec} (Ec), *S. enterica* serovar Typhimurium POTRA chimera (StE), *P. aeruginosa* POTRA chimera (PaE), *P. multocida* POTRA chimera (PmE), *A. tumefaciens* POTRA chimera (AtE), *N. meningitidis* POTRA chimera (NmE), *H. pylori* POTRA chimera (HpE) and *H. influenzae* POTRA chimera (HiE).

Fig. S5. Rescue of BamA depletion by POTRA chimera constructs on agar plates.

A. Growth of JWD3 cells on nutrient agar pates in the presence or absence of arabinose, whilst carrying BamA POTRA chimeras cloned into pASK. Constructs were as follows: *E. coli* BamA_{Ec} (Ec), *H. influenzae* POTRA chimera (HiE), *P. aeruginosa* POTRA chimera (PaE) and *N. meningitidis* POTRA chimera (NmE).

B. Growth of JWD3 cells on nutrient agar plates in the presence or absence of arabinose, whilst carrying the *N. meningitidis* POTRA chimera His-BamA_{NmE} (NmE) cloned into pASK. The R370C, R388G and E521G substitutions enable the His-BamA_{NmE} chimera construct to rescue BamA depletion on agar plates.

C. Growth of JWD3 cells on nutrient agar pates in the presence or absence of arabinose, whilst carrying various *P. aeruginosa* POTRA chimeras cloned into pASK.

D. Growth of JWD3 cells on nutrient agar pates in the presence or absence of arabinose, whilst carrying the *P. aeruginosa* $BamA_{Pa1-4}$ POTRA chimera cloned into pASK. The E470G, D614G, D614N, A654T and D746N substitutions enable $BamA_{Pa1-4}$ to rescue BamA depletion.

Fig. S6. Configuration of loop L6 in BamA orthologues.

A. B-factor putty representation of loop L6 in the available BamA structures. Each structure is shown in a different colour: *N. gonorrhoeae* (4K3B) is in gray, *Haemophilus ducreyi* (4K3C) is salmon pink, *E. coli* (4N75) chains A and B are magenta and orange, respectively, whilst the *E.coli* (4C4V) chains A and B are green and cyan, respectively (Noinaj *et al.*, 2013; Albrecht *et al.*, 2014; Ni *et al.*, 2014). The VGRF motif is coloured blue.

B. Structural super-positioning of the intra-barrel section of loop L6 in different structures (*E.coli* residues 645-670 and the equivalent residues from *N. gonorrhoeae* and *H. ducreyi*), performed using Gesamt from CCP4 Suite (Winn *et al.*, 2011). Chains are coloured as in panel A.

Supplementary Information References.

Albrecht, R., Schutz, M., Oberhettinger, P., Faulstich, M., Bermejo, I., Rudel, T., *et al.* (2014) Structure of BamA, an essential factor in outer membrane protein biogenesis. *Acta Crystallogr D Biol Crystallogr* **70**: 1779-1789.

Browning, D.F., Matthews, S.A., Rossiter, A.E., Sevastsyanovich, Y.R., Jeeves, M., Mason, J.L., *et al.* (2013) Mutational and Topological Analysis of the *Escherichia coli* BamA Protein. *PloS One* **8**: e84512.

Camps, M. (2010) Modulation of ColE1-like plasmid replication for recombinant gene expression. *Rec Pat DNA Gene Seq* **4**: 58-73.

Fleckenstein, J.M., Roy, K., Fischer, J.F. and Burkitt, M. (2006) Identification of a two-partner secretion locus of enterotoxigenic *Escherichia coli*. *Infect Immun* **74**: 2245-2258.

Lehr, U., Schutz, M., Oberhettinger, P., Ruiz-Perez, F., Donald, J.W., Palmer, T., *et al.* (2010) C-terminal amino acid residues of the trimeric autotransporter adhesin YadA of *Yersinia enterocolitica* are decisive for its recognition and assembly by BamA. *Mol Microbiol* **78**: 932-946.

Ni, D., Wang, Y., Yang, X., Zhou, H., Hou, X., Cao, B., *et al.* (2014) Structural and functional analysis of the beta-barrel domain of BamA from *Escherichia coli*. *FASEB J* **28**: 2677-2685.

Noinaj, N., Kuszak, A.J., Gumbart, J.C., Lukacik, P., Chang, H., Easley, N.C., *et al.* (2013) Structural insight into the biogenesis of beta-barrel membrane proteins. *Nature* **501**: 385-390.

Winn, M.D., Ballard, C.C., Cowtan, K.D., Dodson, E.J., Emsley, P., Evans, P.R., *et al.* (2011) Overview of the CCP4 suite and current developments. *Acta Crystallogr D Biol Crystallogr* **67**: 235-242.

Fig. S1

A

>*bamA*$_{Ec}$

CATATGGCGATGAAAAAACTGCTGATCGCGTCTCTGCTGTTCTCTTCTGCGACCGTTTACGGT**GCTAGC**GAAGGTTTCGTTGT
TAAAGACATCCACTTTGAAGGTCTGCAACGTGTTGCGGTTGGTGCGGCGCTGCTGTCTATGCCGGTTCGTACCGGCGACACCG
TGAACGACGAAGACATCTCTAACACCATCCGTGCGCTGTTCGCGACCGGCAACTTTGAAGACGTTCGTGTTCTGCGTGACGGT
GACACCCTGCTGGTTCAGGTTAAAGAACGTCCGACCATCGCGTCTATCACCTTCTCTGGTAACAAATCTGTTAAAGACGACAT
GCTGAAACAGAACCTGGAAGCGTCTGGTGTTCGTGTTGGTGAATCTCTGGACCGTACCACCATCGCGGACATCGAAAAAGGTC
TGGAAGACTTCTACTACTCTGTTGGTAAATACTCTGCGTCTGTTAAAGCGGTTGTTACCCCGCTGCCGCGTAACCGTGTTGAC
CTGAAACTGGTTTTCCAGGAAGGTGTTTCTGCGGAAATCCAGCAGATCAACATCGTTGGTAACCACGCTTTCACCACCGACGA
ACTGATCTCTCACTTCCAACTGCGTGACGAAGTTCCGTGGTGGAACGTGGTTGGTGACCGTAAATACCAGAAACAGAAACTGG
CGGGTGACCTGGAAACCCTGCGTTCTTACTACCTGGACCGTGGTTACGCGCGTTTCAACATCGACTCTACCCAGGTTTCTCTG
ACCCCGGACAAAAAAGGTATCTACGTTACCGTGAACATCACCGAAGGTGACCAGTACAAACTGTCTGGTGTTGAAGTTTCTGG
TAACCTGGCGGGTCACTCTGCGGAAATCGAACAACTGACCAAAATCGAACCGGGTGAACTGTATAACGGCACCAAAGTTACCA
AAATGGAAGACGACATCAAAAAACTGCTGGGTCGTTACGGTTACGCTTACCCGCGTGTTCAGTCTATGCCGGAAATCAACGAC
GCGGACAAAACCGTTAAACTGCGTGTGAACGTGGACGCGGGTAACCGTTTCTACGTTCGTAAAATCCGTTTTGAAGGTAACGA
CACCTCTAAAGACGCGGTTCTGCGTCGTGAAATGCGTCAGATGGAAGGTGCGTGGCTGGGTTCTGACCTGGTTGACCAGGGTA
AAGAACGTCTGAACCGTCTGGGTTTCTTTGAAACCGTTGACACCGACACCCAGCGTGTTCCGGGTTCCCCGGACCAGGTTGAC
GTTGTTTACAAAGTTAAAGAACGTAACACC**GGATCC**TTCAACTTCGGTATCGGTTACGGCACCGAATCTGGTGTTTCTTTCCA
GGCGGGTGTTCAGCAGGACAACTGGCTGGGCACCGGTTACGCGGTTGGTATCAACGGCACCAAAAACGACTACCAGACCTACG
CGGAACTGTCTGTTACCAACCCGTACTTCACCGTTGACGGTGTTTCTCTGGGTGGTCGTCTGTTCTACAACGACTTCCAGGCG
GACGACGCGGACCTGTCTGACTACACCAACAAATCTTACGGCACCGACGTTACCCTGGGTTTCCCGATCAACGAATACAACTC
TCTGCGTGCGGGTCTGGGTTACGTTCACAACTCTCTGTCTAACATGCAGCCGCAGGTTGCGATGTGGCGTTACCTGTACTCTA
TGGGTGAACACCCGTCTACCTCTGACCAGGACAACTCTTTCAAAACCGACGACTTCACCTTCAACTACGGTTGGACCTACAAC
AAACTGGACCGTGGTTACTTCCCGACCGACGGTTCTCGTGTGAACCTGACCGGCAAAGTTACCATTCCGGGTTCTGACAACGA
ATACTACAAAGTTACCCTGGACACCGCGACCTACGTTCCGATTGACGACGACCACAAATGGGTTGTTCTGGGTCGTACCCGTT
GGGGTTACGGTGACGGTCTGGGTGGTAAAGAAATGCCGTTCTACGAAAACTTCTACGCGGGTGGTTCTTCTACCGTTCGTGGT
TTCCAGTCTAACACCATCGGTCCGAAAGCGGTTTACTTCCCGCACCAGGCGTCTAACTACGACCCGGACTACGACTACGAATG
CGCGACCCAGGACGGTGCGAAAGACCTGTGCAAATCTGACGACGCGGTTGGTGGTAACGCGATGGCGGTTGCGTCTCTGGAAT
TTATCACCCCGACCCCGTTCATCTCTGACAAATACGCGAACTCTGTTCGTACCTCTTTCTTCTGGGACATGGGCACCGTTTGG
GACACCAACTGGGACTCTTCTCAGTATTCTGGTTACCCGGACTACTCTGACCCGTCTAACATCCGTATGTCTGCGGGTATCGC
GCTGCAATGGATGTCTCCGCTGGGGCCCCTGGTTTTCTCTTACGCGCAGCCGTTCAAAAAATACGACGGTGACAAAGCGGAAC
AGTTCCAGTTCAACATCGGTAAAACCTGGTAATGAGGGCCCTTAGTGTTTAGCTATGCTCAACCTTTTAAGAAGTATGATGGC
GATAAGGCTGAGCAATTTCAATTTAATATCGGCAAGACTTGGGGCGGCAGATCT**CTCGAG**

>*bamA*$_{St}$

CATATGGCGATGAAAAAACTGCTGATCGCGTCTCTGCTGTTCTCTTCTGCGACCGTTTACGGT**GCTAGC**GAAGGTTTCGTTGT
TAAAGACATCCACTTTGAAGGTCTGCAACGTGTTGCGGTTGGTGCGGCGCTGCTGTCTATGCCGGTTCGTACCGGCGACACCG
TGAACGACGAAGACATCTCTAACACCATCCGTGCGCTGTTTGCGACCGGCAACTTTGAAGACGTTCGTGTTCTGCGTGACGGT
AACACCCTGCTGGTTCAGGTTAAAGAACGTCCGACCATCGCGTCTATCACCTTCTCTGGTAACAAATCTGTTAAAGACGACAT
GCTGAAACAGAACCTGGAAGCGTCTGGTGTTCGTGTTGGTGAATCTCTGGACCGTACCACCCTGTCTGACATCGAAAAAGGTC
TGGAAGACTTCTACTACTCTGTTGGTAAATACTCTGCGTCTGTTAAAGCGGTTGTTACCCCGCTGCCGCGTAACCGTGTTGAC
CTGAAACTGGTTTTCCAGGAAGGTGTTTCTGCGAAAATCCAGCAGATCAACATCGTTGGTAACCACGCTTTCTCTACCGAAGA
ACTGATCTCTCACTTCCAACTGCGTGACGAAGTTCCGTGGTGGAACGTGGTTGGTGACCGTAAATACCAGAAACAGAAACTGG
CGGGTGACCTGGAAACCCTGCGTTCTTACTACCTGGACCGTGGTTACGCGCGTTTCAACATCGACTCTACCCAGGTTTCTCTG
ACCCCGGACAAAAAAGGTATCTACATCACCGTGAACATCACCGAAGGTGACCAGTACAAACTGTCTGGTGTTCAGGTTTCTGG
TAACCTGGCGGGTCACTCTGCGGAAATCGAAAACCTGACCAAAATCGAACCGGGTGAACTGTACAACGGCACCAAAGTTACCA
AAATGGAAGACGACATCAAAAAACTGCTGGGTCGTTACGGTTACGCTTACCCGCGTGTTCAGTCTCAGCCGGAAATCAACGAC
GCGGACAAAACCGTTAAACTGCGTGTGAACGTGGACGCGGGTAACCGTTTCTACGTTCGTAAAATCCGTTTTGAAGGTAACGA
CACCTCTAAAGACTCTGTTCTGCGTCGTGAAATGCGTCAGATGGAAGGTGCGTGGCTGGGTTCTGACCTGGTTGACCAGGGTA
AAGAACGTCTGAACCGTCTGGGTTTCTTTGAAACCGTTGACACCGACACCCAGCGTGTTCCGGGTTCTCCAGACCAGGTTGAC
GTTGTTTACAAAGTTAAAGAACGTAACACC**GGATCC**TTCAACTTCGGTATCGGTTACGGCACCGAATCTGGTGTTTCTTTCCA
GGCGGGTGTTCAGCAGGACAACTGGCTGGGCACCGGCTACTCTGTTGGTATCAACGGCACCAAAAACGACTACCAGACCTACT
CTGAACTGTCTGTTACCAACCCGTACTTCACCGTTGACGGTGTTTCTCTGGGTGGTCGTATCTTCTACAACGACTTCCAGGCG
GACGACGCGGACCTGTCTGACTACACCAACAAATCTTACGGCACCGACGTTACCCTGGGTTTCCCGATCAACGAATACAACAC
CCTGCGTGCGGGTCTGGGTTACGTTCACAACAAACTGTCTAACATGCAGCCGCAGATCGCTATGGACCGTTACCTGGAATCTA
TGGGTCAGTCTGCGGACACCTCTTCTTTCGCGGCGGACGACTTCACCTTCAACTACGGTTGGACCTACAACAAACTGGACCGT
GGTTACTTCCCGACCGACGGTTCTCGTGTGAACCTGACCGGCAAAGTTACCATCCCAGGTTCTGACAACGAATACTACAAAGT
TTCTCTGGACACCGCGACCTACGTTCCGATTGACAACGACCACAAATGGGTTGTTCTGGGTCGTACCCGTTGGGGTTACGGTG
ACGGTCTGGGTGGTAAAGAAATGCCGTTCTACGAAAACTTCTACGCGGGTGGTTCTTCTACCGTTCGTGGTTTCCAGTCTAAC
ACCATCGGTCCGAAAGCGGTTTACAAAAACGGTGCTCACACCTCTTGGGACGACAACGACGACTACGAAGACTGCACCCAGGA

ATCTGGTTGCAAATCTGACGACGCGGTTGGTGGTAACGCGATGGCGGTTGCGTCTCTGGAATTTATCACCCCGACCCCGTTCA
TCTCTGAAAAATACGCGAACTCTGTTCGTACCTCTTTCTTCTGGGACATGGGCACCGTTTGGGACACCAACTGGGACCCGTCT
TCTGCGCCGTCTGACGTTCCAGACTACTCTGACCCAGGTAACATCCGTATGTCTGCGGGTATCGCGCTGCAATGGATGTCTCC
GCTGGGGCCCCTGGTTTTCTCTTACGCGCAGCCGTTCAAAAAATACGACGGTGACAAAGCGGAACAGTTCCAGTTCAACATCG
GTAAAACCTGGTAATGAGGGCCCTTAGTGTTTAGCTATGCTCAACCATTTAAGAAGTATGATGGCGATAAGGCTGAGCAATTT
CAATTTAATATCGGCAAGACTTGGGGCGGCAGATCT**CTCGAG**

>*bamA*$_{Pa}$
CATATGGCGATGAAAAAACTGCTGATCGCGTCTCTGCTGTTCTCTTCTGCGACCGTTTACGGT**GCTAGC**GAATCTTTCACCGT
TTCTGACATCCGTGTGAACGGTCTGCAACGTGTTTCTGCGGGTTCTGTTTTCGCGGCGCTGCCGCTGAACGTGGGTGAAACCA
TCGACGACCAGGCGCTGGTTCAGGCGACCCGTTCTCTGTTCAAAACCGGCTTCTTCCAGGACATCCAACTGGGTCGTGACGGT
AACGTGCTGGTTGTTACCGTTGTTGAACGTCCGTCTATCTCTTCTATCGAAATCGAAGGTAACAAAGCGATCTCTAAAGAAGA
CCTGCTGAAAGGTCTGAAACAGTCTGGTCTGGCGGAAGGTGAAATCTTCCAGCGTGCGACCCTGGAAGGTGTTCGTAACGAAC
TGCAACGTCAGTACGTTGCTCAGGGTCGTTACTCTGCGGAAATCAACGCGGAAGTTATCCCGCAGCCGCGTAACCGTGTTGCG
CTGAAAATCAACATCAACGAAGGCACCGTTGCGGCGATCTCTCACATCAACGTGGTTGGTAACACCGTTTTCTCTGAAGAAGA
CCTGACCGACCTGTTTGAACTGAAAACCACCAACTGGCTGTCTTTCTTCAAAAACGACGACAAATACGCGCGTGAAAAACTGT
CTGGTGACCTGGAACGTCTGCGTTCTTACTACCTGGACCGTGGTTACATCAACATGGACATCGCGTCTACCCAGGTTTCTATC
ACCCCGGACAAAAAACACGTTTACATCACCGTGAACATCAACGAAGGTGAAAAATACACCATCCGTGACGTTAAACTGACCGG
CGACCTGAAAGTGCCGGAAGAAGAAGTTAAACGTCTGCTGCTGGTTCAGAAAGGTCAGGTTTTCTCTCGTAAAGTTATGACCA
CCACCTCTGACCTGATTACCCGTCGTCTGGGTAACGAAGGTTACACCTTTGCGAACGTGAACGGTGTGCCGGAAGCGCACGAC
GACGACAAAACCGTTTCTGTTACCTTCGTTGTTGACCCAGGTAAACGTGCGTATGTGAACCGTATCAACTTCCGTGGTAACAC
CAAAACCGAAGACGAAGTTCTGCGTCGTGAAATGCGTCAGATGGAAGGTGGTTGGGCGTCTACCTACCTGATCGACCAGTCTA
AAGCGCGTCTGGAACGTCTGGGTTACTTCAAAGAAGTGAACGTGGAAACCCCGGCGGTTCCGGGCACCGACGACCAGGTTGAC
GTGAACTACTCTGTTGAAGAACAGCCGTCT**GGATCC**ATCACCGCGTCTGTTGGTTTCGCGCAGTCTGCGGGTCTGATCCTGGG
TGGTTCTATCTCTCAGAACAACTTCCTGGGCACCGGCAACAAAGTTTCTATCGGTCTGACCCGTTCTGAATACCAGACCCGTT
ACAACTTCGGTTTCGTTGACCCGTACTGGACCGTTGACGGTGTTTCTCTGGGTTACAACGCTTTCTACCGTAAAACCGACTAC
GACGAACTGGACGTTGACGTTGCGTCTTACTCTGTGAACTCTCTGGGTGCGGGTATGTCTATCGGTTACCCGATCTCTGAAAC
CTCTCGTCTGACCTACGGTCTGTCTGTTCAGCGTGACCAGATCGACACTGGCCGTTACACCGTTGACGAAATCTACGACTTCC
TGGACAAAGAAGGTGACAACTTCACCAACTTCAAAGCGTCTATCGGTTGGTCTGAATCTACCCTGAACAAAGGTGTTCTGGCG
ACCCGTGGTCACTCTCAGTCTCTGACCCTGGAAACCACCCTGCCGGGTTCTGACCTGTCTTTCTACAAAATCGACTACCGTGG
TCAGGTTTTCGCGCCGCTGACCGACAACTACACCATGCGTTTCCACACCGAACTGGGTTACGGTGACGGTTACGGTTCTACCG
AACGTCTGCCGTTCTACGAAAACTACTACGCGGGTGGTTTCAACTCTGTTCGTGGTTTCAAAGACTCTACCCTGGGTCCGCGT
TCTACCCCGTCTGTTGCGCGTAACCCGGACGGCACCCCGATGAAAAACCAGGGTCCAGACTCTAAAGGTCGTTACACCGACCC
GGACCAGGACCCGGAAGCGTTCGGTGGTAACATCCTGATTACCGGCGGTGCGGAACTGCTGTTCCCGCTGCCGTTCGTTAAAG
ACCAGCGTCAACTGCGTACCGTTCTGTTCTGGGACGTTGGTTCTACCTTCGACACCGACTGCCCGACCAAAACCACCACCAAC
TGCGACGGTATCAAAACCGACAACCTGGCGTCTTCTGTTGGTGTTGGTCTGACCTGGATCACCGCGCTGGGGCCCCTGTCTTT
CTCTCTGGCGACCCCGATCAAAAAACCGGACAACGCGGAAACCCAGGTTTTCCAGTTCTCTCTGGGTCAGACCTTCTAATGAG
GGCCCTTAAGATTTAGATTAGCTACTCCAATTAAGAAGCCAGATAATGCTGAGACTCAAGTGTTTCAATTTAGATTAGGCCAA
ACTTTTGGCGGCAGATCT**CTCGAG**

>*bamA*$_{Pm}$
CATATGGCGATGAAAAAACTGCTGATCGCGTCTCTGCTGTTCTCTTCTGCGACCGTTTACGGT**GCTAGC**CCGTTCGTTGTTAA
AGACATCCGTGTTGACGGTGTTCAGGCGGGCACCGAAGGTTCTGTTCTGGCGACCCTGCCGGTTCGTGTTGGTCAGCGTGCGA
CCGACAACGACATCGCTAACGTGGTTCGTAAACTGTTCCTGTCTGGTCAGTACGACGACGTTAAAGCGTCTCGTGAAGGTAAC
ACCCTGGTTGTTACCGTTATGCCGAAACCAGTTATCTCTAACGTGGTTATCGTTGGTAACAAATCTATCCCGGACGAAGCGAT
CAAACAGAACCTGGACGCGAACGGTTTCAAAGTTGGTGACGTTCTGAACCGTGCGAAACTGGAAGAATTTCGTAAAGGTATCG
TTGAACACTACAACTCTGTTGGTCGTTACAACGCGAAAGTTGACGCTATCGTGAACACCCTGCCGAACAACTCTGCGGAAATC
AAAATCCAGATCAACGAAGACGACGTTGCGCTGTTCAAAGAAATCACCTTTGAAGGTAACGAAGCGTTCTCTTCTGGTAAACT
GGCGGACCAGATGGAACTGCAAACCGACTCTTGGTGGAAACTGTTCGGTAACAAATTCGACCAGACCCAGTTCAACAAAGACC
TGGAAACCCTGCGTTCTTACTACCTGGACCGTGGTTACGCGCAGTTCCAGATCCTGGACACCGACGTTAAACTGTCTGACGAC
AAAAAAGAACCGTGCCTGATCTCTGAAGAAGGTGACCTGTACACCGTTAAAACCCGTGTTTCTGGTGGTATGTGGGGTGGTAT
GTCTGCGGAACTGGCGCCGATCCTGGAAACCATCCAACTGAACGGTCTGTTCCGTCGTACCTCTGTTCTGGAAGTTGAACAGC
GTAACAAATCTAAACTGGGTGAACGTGGTTACGCGACCGCGCAGGTGAACGTGCATCCGACCTTCGACGAACAGGACAAAACC
ATCTCTCTGGACTTCATCGTTGAAGCGGGTAAATCTTACACCGTTCGTCAGATCCGTTTTGAAGGTAACACCTCTTCTGCGGA
CTCTACCCTGCGTCAGGAAATGCGTCAGCAGGAAGGTGCGTGGCTGTCTTCTGAACTGGTTGAACTGGGTAAACTGCGTCTGG
ACCGTACCGGCTACTTTGAATCTGTTGAAACCAAAACCGAAGCGATCCCAGGTTCTGACCAGGTTGACGTTATCTACAAAGTT
AAAGAACGTAACACC**GGATCC**ATCAACTTCGGTATCGGTTACGGCACCGAATCTGGTCTGTCTTACCAGGCGTCTATCAAACA
GGACAACTTCCTGGGTATGGGTTCTTCTATCTCTCTGGGTGGCACCCGTAACGACTACGGCACCACCGTGAACCTGGGTTACA
ACGAACCGTACTTCACCAAAGACGGTGTTTCTCTGGGTGGTAACGTGTCTTTTGAAGAATACGACTCTTCTAAATCTAACACC
TCTGCGGGTTACGGTCGTACCTCTTACGGTGGTAACCTGACCCTGGGTTTCCCGGTGAACGAAAACAACTCTTACTACCTGGG
TGTTGGTTACACCTACAACAAACTGAAAAACATCGCGCCGGAATACAACCGTGACCTGTACCGTCAGTCTATGAAATACAACG
ACTCTTGGACCTTCAAATCTCACGACTTCGACCTGTCTTTCGGTTGGAACTACAACTCTCTGAACCGTGGTTACTTCCCGACC
AAAGGTGTTCGTGCGAACATCGGTGGTCGTGTTACCATCCCAGGTTCTGACAACAAATACTACAAACTGAACGCGGAAGCGCA
GGGTTTCTACCCGCTGGACCGTGAACACGGTTGGGTTCTGTCTTCTCGTATCTCTGCGTCTTTCGCGGACGGTTTCTCTGGTA
AACGTCTGCCGTTCTACCAGTACTACTCTGCGGGTGGTATCGGTTCTCTGCGTGGTTTCGCGTATGGTGCTATCGGTCCGAAC
GCGATCTACCGTACCCGTCAGTGCCCGGACTCTTACTGCCTGGTTTCTTCTGACGTTATCGGTGGTAACGCGATGGTTACCGC

GTCTACCGAACTGATCGTTCCGACCCCGTTCGTTGCGGACAAAAACCAGAACTCTGTTCGTACCTCTCTGTTCGTTGACGCGG
CGTCTGTTTGGAACACCCGTTGGAAAGCGGAAGACAAAGCGAAATTTGCGAAACTGAACGTGCCGGACTACTCTGACCCGTCT
CGTGTTCGTGCGTCTGCGGGTGTTGCGCTGCAATGGCAGTCTCCGATTGGGCCCCTGGTTTTCTCTTACGCGAAACCGCTGAA
AAAATACCAGGGTGACGAAATCGAACAGTTCCAGTTCTCTATCGGTGGCACCTTCTAATGAGGGCCCTTAGTGTTTAGCTATG
CTAAGCCATTAAAGAAGTATCAAGGCGATGAGATTGAGCAATTTCAATTTAGCATTGGCGGCACTTTTGGCGGCAGATCT**CTC
GAG**

>*bamA*$_{At}$
CATATGGCGATGAAAAAACTGCTGATCGCGTCTCTGCTGTTCTCTTCTGCGACCGTTTACGGT**GCTAGC**GTTATCTCTAAAAT
CGACGTTCGTGGTGCGGAGCGTTCTGGTGCGGACTCTGTTCGTTCTAACATCACCATCGCGCCGGGTAAAAACTTCTCTAACT
CTGACATCGACGAATCTGTTAAACGTCTGTACGCGACCGGCTACTTCTCTAACGTGTCTATGCGTGTTTCTGGTTCTACCCTG
GTTGTTACCGTGAACGAAAACCAACTGGTGAACCAGGTTGTTTTCAACGGTAACCGTAAAATCAAAGACGACAAACTGGCGGG
TATCGTTCAGACCCAGCCGATGGGTCCGTTCAACCAGGCTATCGTTACCGCTGACATCGCGCGTATCAAAGAAGCGTACTCTG
CTATCGGTCGTTCTGACGTTGAAATCACCACCCAGACCGTTTCTGTTGGTCAGGGTCGTGTGAACATCGCGTTCGTTATCAAC
GAAGGTGAACGTACCAAAATCGGTCGTATCGACTTCATCGGTAACAACTCTTACTCTGACGGTCGTCTGGCGGCGGTTATCAA
CACCAAAAAATCTAACATGCTGTCTTTCCTGACCCGTAAAGACGTTTACAACGAAGACAAACTGCGTGCGGACGAAGAAGCTC
TGCGTCAGTTCTACTACAACCGTGGTTACGCGGACTTCCGTGTTGTTTCTTCTGACGCGGTTCTGGACGAATCTAAAAACGAA
TACACCATCTCTATCACCGTTGACGAAGGTAAAAAATACGACTTCGGTAACGTGGCGGTTGAATCTACCGTTCCGGGTGTTGA
CGGTTCTGAACTGCAAGGTCTGGTTGAAACCCGTCAGGGTGCGTCTTACTCTGCGAAAGAAGTTCAGCAGTCTATGGAAGCGA
TCTCTAAACGTGTTGCGGGTGAAGGTTACCCGTTCGCGCGTGTTACCCCGCGTGGTGACCGTGATATGTCTGGTAACACCATC
GGTGTTACCTACATCGTTGACCAGGGTGAACGTGCGTATGTTGAACGTATCGAAATCCGTGGTAACACCCGTACCCGTGACTA
CGTTATCCGTCGTGAATTTGACATCTCTGAAGGTGACGCTTTCAACCAGACCATCATCACCGCTGCGAAACGTCGTCTGGAAG
CTCTGGGTTACTTCTCTAAAGTGAACATCTCTACCGCTGGTGGTTCTGCGCCGGACCGTGTTGTTATCGTTGTTGACGTTGAA
GACCAGTCTACC**GGATCC**TTCGGTATCGGTGCGGGTTACTCTCAGAACGACGGTGTTCTGCTGGAAGCGTCTGTTGAAGAAAA
AAACTTCCTGGGTCGTGGTCAGTACATCCGTGTTGCGGCGGGTGCGGGTGAAGACGACGCGCGTACCTACTCTCTGTCTTTCA
CCGAACCGTACTTCCTGGGTTACCGTCTGGCGGCGGGTTTCGACCTGTTCAAAAACCAGTCTAAATCTGAAGACTACTACAAC
TACGACGAACAGGGTTTCGCGCTGCGTGTTACCGCGCCGATCACCGAAAACCTGTCTACCACCTTCAAATACACCTACAAACA
GATCAACTACGAAGGTAAAGGTGACTGGCAGAACAACGCGAACCTGGCGGAACCGTACCAGGCGCTGATCCGTGGTGAAGACT
GGACCCAGTCTATCCTGTCTAACACCCTGAACTACAACACCCTGGACGACCGTAACATGCCGCGTGAAGGTTGGCAGGCGGCG
CTGACCAACGAATTTGCGGGTCTGGGTGGTGACTCTGAATACTACAAAATCTACGCGAAAGCGCGTTACTACTACACCCTGTC
TGACGAATACGACGTTATCGGTTCTCTGACCGGCCAGGCGGGTCACGTTATGCCGACCGGCGACAACCTGCTGGTTTTCGACC
AGTTCAAATTCGGTGGTCGTCAGGTTCGTGGTTTCAAAAACGACGGTATCGGTCCGCGTATCGGTTCTGACTCTATCGGTGGC
ACCACCTACTTCGCGGCGTCTGCGGAAGTTACCGCGCCGATGCCGGGTGTGCCGGAAGACTTCGGTCTGCGTCTGGCGGGTTT
CGTTGACGCGGGCACCATGTACGGTAACAAAGTTTCTACCTCTCAGACCGTTAAAGACGACAACTCTATCCGTGCGTCTGCGG
GTATCGGTGTTATGTGGGCGTCTCCGTTCGGGCCCATCCGTGTTGACTACGCGATCCCGATTGCGAAAGAAGACTACGACGAA
GAACAGCGTTTCCGTTTCGGTATGTCTAACACCTTCTAATGAGGGCCCATTAGAGTGGATTATGCTATTCCAATCGCAAAGGA
GGATTATGATGAGGAGCAAAGATTTAGATTTGGCATGAGCAATACTTTTGGCGGCAGATCT**CTCGAG**

>*bamA*$_{Nm}$
CATATGGCGATGAAAAAACTGCTGATCGCGTCTCTGCTGTTCTCTTCTGCGACCGTTTACGGT**GCTAGC**GACTTCACCATCCA
GGACATCCGTGTTGAAGGTCTGCAACGTACCGAACCGTCTACCGTTTTCAACTACCTGCCGGTTAAAGTTGGTGACACCTACA
ACGACACCCACGGTTCTGCGATCATCAAATCTCTGTACGCGACCGGCTTCTTCGACGACGTTCGTGTTGAAACCGCTGACGGT
CAACTGCTGCTGACCGTTATCGAACGTCCGACCATCGGTTCTCTGAACATCACCGGCGCGAAAATGCTGCAAAACGACGCGAT
CAAAAAAAACCTGGAATCTTTCGGTCTGGCGCAGTCTCAGTATTTCAACCAGGCGACCCTGAACCAGGCGGTTGCGGGTCTGA
AAGAAGAATACCTGGGTCGTGGTAAACTGAACATCCAGATCACCCCGAAAGTTACCAAACTGGCGCGTAACCGTGTTGACATC
GACATCACCATCGACGAAGGTAAATCTGCGAAAATCACCGACATCGAATTTGAAGGTAACCAGGTTTACTCTGACCGTAAACT
GATGCGTCAGATGTCTCTGACCGAAGGTGGTATCTGGACCTGGCTGACCCGTTCTAACCAGTTCAACGAACAGAAATTCGCGC
AGGACATGGAAAAAGTTACCGACTTCTACCAGAACAACGGTTACTTCGACTTCCGTATCCTGGACACCGACATCCAGACCAAC
GAAGACAAAACCAAACAGACCATCAAAATCACCGTTCACGAAGGTGGTCGTTTCCGTTGGGGTAAAGTTTCTATCGAAGGTGA
CACCAACGAAGTTCCGAAAGCGGAACTGGAAAAACTGCTGACCATGAAACCGGGTAAATGGTACGAACGTCAGCAGATGACCG
CTGTTCTGGGTGAAATCCAGAACCGTATGGGTTCTGCGGGTTACGCTTACTCTGAAATCTCTGTTCAGCCGCTGCCGAACGCG
GAAACCAAAACCGTTGACTTCGTTCTGCACATCGAACCGGGTCGTAAAATCTACGTGAACGAAATCCACATCACCGGCAACAA
CAAAACCCGTGACGAAGTTGTTCGTCGTGAACTGCGTCAGATGGAATCTGCGCCGTACGACACCTCTAAACTGCAACGTTCTA
AAGAACGTGTTGAACTGCTGGGTTACTTCGACAACGTGCAGTTCGACGCGGTTCCGCTGGCGGGCACCCCGGACAAAGTTGAC
CTGAATATGTCTCTGACCGAGCGTTCTACC**GGATCC**CTGGACCTGTCTGCGGGTTGGGTTCAGGACACCGGCCTGGTTATGTC
TGCGGGTGTTTCTCAGGACAACCTGTTCGGCACCGGCAAATCTGCGGCGCTGCGTGCGTCTCGTTCTAAAACCACCCTGAACG
GTTCTCTGTCTTTCACCGACCCGTACTTCACCGCTGACGGTGTTTCTCTGGGTTACGACGTTTACGGTAAAGCGTTCGACCCG
CGTAAAGCGTCTACCTCTATCAAACAGTACAAAACCACCACCGCTGGTGCGGGTATCCGTATGTCTGTTCCGGTTACCGAATA
CGACCGTGTGAACTTCGGTCTGGTTGCGGAACACCTGACCGTGAACACCTACAACAAAGCGCCGAAACACTACGCGGACTTCA
TCAAAAAATACGGTAAAACCGACGGCACCGACGGTTCTTTCAAAGGTTGGCTGTATAAAGGCACCGTTGGTTGGGGTCGTAAC
AAAACCGACTCTGCGCTGTGGCCGACCCGTGGTTACCTGACCGGCGTGAACGCGGAAATCGCGCTGCCGGGTTCTAAACTGCA
ATACTACTCTGCGACCCACAACCAGACCTGGTTCTTCCCGCTGTCTAAAACCTTCACCCTGATGCTGGGTGGTGAAGTTGGTA
TCGCGGGTGGTTACGGTCGTACCAAAGAAATCCCGTTCTTTGAAAACTTCTACGGTGGTGGTCTGGGTTCTGTTCGTGGTTAC
GAATCTGGCACCCTGGGTCCGAAAGTTTACGACGAATACGGTGAAAAAATCTCTTACGGTGGTAACAAAAAAGCGAACGTGTC
TGCGGAACTGCTGTTCCCGATGCCGGGTGCGAAAGACGCGCGTACCGTTCGTCTGTCTCTGTTCGCGGACGCGGGTTCTGTTT
GGGACGGTAAAACCTACGACGACAACTCTTCTTCTGCGACCGGCGGTCGTGTTCAGAACATCTACGGTGCGGGTAACACCCAC

AAATCTACCTTCACCAACGAACTGCGTTACTCTGCGGGTGGTGCGGTTACCTGGCTGTCTCCGCTGGGGCCCATGAAATTCTC
TTACGCTTACCCGCTGAAAAAAAAACCGGAAGACGAAATCCAGCGTTTCCAGTTCCAACTGGGCACCACCTTCTAATGAGGGC
CCATGAAGTTTAGCTATGCCTATCCATTAAAGAAGAAGCCAGAGGATGAGATTCAAAGATTTCAATTTCAATTAGGTACTACT
TTTGGCGGCAGATCT**CTCGAG**

>*bamA*$_{Hp}$
CATATGGCGATGAAAAAACTGCTGATCGCGTCTCTGCTGTTCTCTTCTGCGACCGTTTACGGT**GCTAGC**CTGGAAAACGACGG
TTCTAAACCGAACGACCTGACCTCTCCGAAAGAAGCGTCTCAGGAATCTCAGAAAAACGAAGCGCCGAAAAACGAAGTTCAGC
GTAACGAAGCGCAGAAAGAAACCCCGCAGTCTAACCAGACCCCGAAAGAAATGAAAGTTAAATCTATCTCTTACGTTGGTCTG
TCTTATATGTCTGACATGCTGGCGAACGAAATCGTTAAAATCCGTGTTGGTGACATCGTTGACTCTAAAAAAATCGACACCGC
TGTTCTGGCGCTGTTCAACCAGGGTTACTTCAAAGACGTTTACGCGACCTTTGAAGGTGGTATCCTGGAATTTCACTTCGACG
AAAAAGCGCGTATCGCGGGTGTTGAAATCAAAGGTTACGGCACCGAAAAAGAAAAAGACGGTCTGAAATCTCAGATGGGTATC
AAAAAAGGTGACACCTTCGACGAACAGAAACTGGAACACGCGAAAACCGCGCTGAAAACCGCGCTGGAAGGTCAGGGTTACTA
CGGTTCTGTTGTTGAAGTTCGTACCGAAAAAGTTTCTGAAGGTGCGCTGCTGATCGTTTTCGACGTGAACCGTGGTGACTCTA
TCTACATCAAACAGTCTATCTACGAAGGTTCTGCGAAACTGAAACGTCGTATGATCGAATCTCTGTCTGCGAACAAACAGCGT
GACTTCATGGGTTGGATGTGGGGTCTGAACGACGGTAAACTGCGTCTGGACCAACTGGAATACGACTCTATGCGTATCCAGGA
CGTTTACATGCGTCGTGGTTACCTGGACGCGCACATCTCTTCTCCGTTCCTGAAAACCGACTTCTCTACCCACGACGCGAAAC
TGCACTACAAAGTTAAAGAAGGTATCCAGTACCGTATCTCTGACATCCTGATCGAAATCGACAACCCGGTTGTTCCGCTGAAA
ACCCTGGAAAAAGCTCTGAAAGTTAAACGTAAAGACGTTTTCAACATCGAACACCTGCGTGCGGACGCGCAGATCCTGAAAAC
CGAAATCGCGGACAAAGGTTACGCTTTCGCGGTTGTTAAACCGGACCTGGACAAAGACGAAAAAAACGGTCTGGTTAAAGTTA
TCTACCGTATCGAAGTTGGTGACATGGTTTACATCAACGACGTTATCATCTCTGGTAACCAGCGTACCTCTGACCGTATCATC
CGTCGTGAACTGCTGCTGGGTCCGAAAGACAAATACAACCTGACCAAACTGCGTAACTCTGAAAACTCTCTGCGTCGTCTGGG
TTTCTTCTCTAAAGTTAAAATCGAAGAAAAACGTGTGAACTCTTCTCTGATGGACCTGCTGGTTTCTGTTGAAGAAGGTCGTA
CC**GGATCC**CTGCAATTCGGTCTGGGTTACGGTTCTTACGGTGGTCTGATGCTGAACGGTTCTGTTTCTGAACGTAACCTGTTC
GGCACCGGCCAGTCTATGTCTCTGTACGCGAACATCGCTACCGGCGGTGGTCGTTCTTACCCAGGTATGCCGAAAGGTGCGGG
TCGTATGTTCGCGGGTAACCTGTCTCTGACCAACCCGCGTATCTTCGACTCTTGGTACTCTTCTACCATCAACCTGTACGCGG
ACTACCGTATCTCTTACCAGTACATCCAGCAGGGTGGTGGTTTCGGTGTGAACGTGGGTCGTATGCTGGGTAACCGTACCCAC
GTTTCTCTGGGTTACAACCTGAACGTGACCAAACTGCTGGGTTTCTCTTCTCCGCTGTACAACCGTTACTACTCTTCTGTGAA
CGAAGTTGTTTCTCCGCGTCAGTGCTCTACCCCGGCGTCTGTTATCATCAACCGTCTGTCTGGTGGTAAAACCCCGCTGCAAC
CGGAATCTTGCTCTTCTCCGGGTGCGATCACCACCTCTCCAGAAATCCGTGGTATCTGGGACCGTGACTACCACACCCCGATC
ACCTCTTCTTTCACCCTGGACGTTTCTTACGACAACACCGACGACTACTACTTCCCGCGTAACGGTGTTATCTTCTCTTCTTA
CGCGACCATGTCTGGTCTGCCGTCTTCTGGCACCCTGAACTCTTGGAACGGTCTGGGTGGTAACGTGCGTAACACCAAAGTTT
ACGGTAAATTCGCGGCGTACCACCACCTGCAAAAATACCTGCTGATCGACCTGATCGCGCGTTTCAAAACCCAGGGTGGTTAC
ATCTTCCGTTACAACACCGACGACTACCTGCCGCTGAACTCTACCTTCTACATGGGTGGTGTTACCACCGTTCGTGGTTTCCG
TAACGGTTCTGTTACCCCGAAAGACGAATTTGGTCTGTGGCTGGGTGGTGACGGTATCTTCACCGCGTCTACCGAACTGTCTT
ACGGTGTTCTGAAAGCGGCGAAAATGCGTCTGGCGTGGTTCTTCGACTTCGGTTTCCTGACCTTCAAAACCCCGACCCGTGGT
TCTTTCTTCTACAACGCGCCGGTTACCACCGCGAACTTCAAAGACTACGGTGTTATCGGTGCGGGTTTTGAACGTGCGACCTG
GCGTGCGTCTACCGGCCTGCAAATCGAATGGATCTCTCCGATGGGGCCCCTGGTTCTGATCTTCCCGATTGCGTTCTTCAACC
AGTGGGGTGACGGTAACGGTAAAAAATGCAAAGGTCTGTGCTTCAACCCGAACATGGACGACTACACCCAGCACTTTGAATTT
TCTATGGGCACCCGTTTCTAATGAGGGCCCTTAGTGTTAATTTTTCCAATCGCTTTTTTTAATCAATGGGGCGATGGCAATGG
CAAGAAGTGTAAGGGCTTATGTTTTAATCCAAATATGGATGATTATACGCAACATTTTGAGTTTAGCATGGGGACTAGATTTG
GCGGCAGATCT**CTCGAG**

>*bamA*$_{Hi}$
CATATGGCGATGAAAAAACTGCTGATCGCGTCTCTGCTGTTCTCTTCTGCGACCGTTTACGGT**GCTAGC**CCGTTCGTTGCGAA
AGACATCCGTGTTGACGGTGTTCAGGGTGACCTGGAACAGCAGATCCGTGCGTCTCTGCCGGTTCGTGCGGGTCAGCGTGTTA
CCGACAACGACGTTGCGAACATCGTTCGTTCTCTGTTCGTTTCTGGTCGTTTCGACGACGTTAAAGCGCACCAGGAAGGTGAC
GTTCTGGTTGTTTCTGTTGTTGCGAAATCTATCATCTCTGACGTTAAAATCAAAGGTAACTCTATCATCCCGACCGAAGCTCT
GAAACAGAACCTGGACGCGAACGGTTTCAAAGTTGGTGACGTTCTGATCCGTGAAAAACTGAACGAATTTGCTAAATCTGTTA
AAGAACACTACGCTTCTGTTGGTCGTTACAACGCGACCGTTGAACCGATTGTGAACACCCTGCCGAACAACCGTGCGGAAATC
CTGATCCAGATCAACGAAGACGACAAAGCGAAACTGGCGTCTCTGACCTTCAAAGGTAACGAATCTGTTTCTTCTTCTACCCT
GCAAGAACAGATGGAACTGCAACCGGACTCTTGGTGGAAACTGTGGGGTAACAAATTTGAAGGTGCTCAGTTTGAAAAAGACC
TGCAATCTATCCGTGACTACTACCTGAACAACGGTTACGCGAAAGCGCAGATCACCAAAACCGACGTTCAACTGAACGACGAA
AAAACCAAAGTGAACGTGACCATCGACGTGAACGAAGGTCTGCAATACGACCTGCGTTCTGCGCGTATCATCGGTAACCTGGG
TGGTATGTCTGCGGAACTGGAACCGCTGCTGTCTGCGCTGCACCTGAACGACACCTTCCGTCGTTCTGACATCGCGGACGTTG
AAAACGCGATCAAAGCGAAACTGGGTGAACGTGGTTACGGTTCTGCGACCGTGAACTCTGTTCCAGACTTCGACGACGCGAAC
AAAACCCTGGCGATCACCCTGGTTGTTGACGCGGGTCGTCGTCTGACCGTTCGTCAACTGCGTTTTGAAGGTAACACCGTTTC
TGCGGACTCTACCCTGCGTCAGGAAATGCGTCAGCAGGAAGGCACCTGGTACAACTCTCAACTGGTTGAACTGGGTAAAATCC
GTCTGGACCGTACCGGCTTCTTTGAAACCGTTGAAAACCGTATCGACCCGATCAACGGTTCTAACGACGAAGTTGACGTTGTT
TACAAAGTTAAAGAACGTAACACC**GGATCC**ATCAACTTCGGTATCGGTTACGGCACCGAATCTGGTATCTCTTACCAGGCGTC
TGTTAAACAGGACAACTTCCTGGGCACCGGCGCGGCGGTTTCTATCGCGGGCACCAAAAACGACTACGGCACCTCTGTGAACC
TGGGTTACACCGAACCGTACTTCACCAAAGACGGTGTTTCTCTGGGTGGTAACGTGTTCTTTGAAAACTACGACAACTCTAAA
TCTGACACCTCTTCTAACTACAAACGTACCACCTACGGTTCTAACGTGACCCTGGGTTTCCCGGTGAACGAAAACAACTCTTA
CTACGTTGGTCTGGGTCACACCTACAACAAAATCTCTAACTTCGCGCTGGAATACAACCGTAACCTGTACATCCAGTCTATGA
AATTCAAAGGTAACGGTATCAAAACCAACGACTTCGACTTCTCTTTCGGTTGGAACTACAACTCTCTGAACCGTGGTTACTTC
CCGACCAAAGGTGTTAAAGCGTCTCTGGGTGGTCGTGTTACCATCCCAGGTTCTGACAACAAATACTACAAACTGTCTGCGGA

CGTTCAGGGTTTCTACCCGCTGGACCGTGACCACCTGTGGGTTGTTTCTGCGAAAGCGTCTGCGGGTTACGCGAACGGTTTCG
GTAACAAACGTCTGCCGTTCTACCAGACCTACACCGCTGGTGGTATCGGTTCTCTGCGTGGTTTCGCGTATGGTTCTATCGGT
CCGAACGCGATCTACGCGGAACACGGTAACGGTAACGGCACCTTCAAAAAAATCTCTTCTGACGTTATCGGTGGTAACGCGAT
CACCACCGCGTCTGCGGAACTGATCGTTCCGACCCCGTTCGTTTCTGACAAATCTCAGAACACCGTTCGTACCTCTCTGTTCG
TTGACGCGGCGTCTGTTTGGAACACCAAATGGAAATCTGACAAATCTGGTCTGGACAACAACGTGCTGAAATCTCTGCCGGAC
TACGGTAAATCTTCTCGTATCCGTGCGTCTACCGGCGTTGGTTTCCAGTGGCAGTCTCCGATTGGGCCCCTGGTTTTCTCTTA
CGCGAAACCGATCAAAAAATACGAAAACGACGACGTTGAACAGTTCCAGTTCTCTATCGGTGGTTCTTTCTAATGAGGGCCCT
TAGTGTTTAGCTATGCTAAGCCTATTAAGAAGTATGAGAATGATGATGTGGAGCAATTTCAATTTAGCATTGGCGGCAGCTTT
GGCGGCAGATCT**CTCGAG**

>*bamA*$_{EtpB}$
CATATGGCGATGAAAAAACTGCTGATCGCGTCTCTGCTGTTCTCTTCTGCGACCGTTTACGGT**GCTAGC**GAAGGTTTCGTTGT
TAAAGACATCCACTTTGAAGGTCTGCAACGTGTTGCGGTTGGTGCGGCGCTGCTGTCTATGCCGGTTCGTACCGGCGACACCG
TGAACGACGAAGACATCTCTAACACCATCCGTGCGCTGTTCGCGACCGGCAACTTTGAAGACGTTCGTGTTCTGCGTGACGGT
GACACCCTGCTGGTTCAGGTTAAAGAACGTCCGACCATCGCGTCTATCACCTTCTCTGGTAACAAATCTGTTAAAGACGACAT
GCTGAAACAGAACCTGGAAGCGTCTGGTGTTCGTGTTGGTGAATCTCTGGACCGTACCACCATCGCGGACATCGAAAAAGGTC
TGGAAGACTTCTACTACTCTGTTGGTAAATACTCTGCGTCTGTTAAAGCGGTTGTTACCCCGCTGCCGCGTAACCGTGTTGAC
CTGAAACTGGTTTTCCAGGAAGGTGTTTCTGCGGAAATCCAGCAGATCAACATCGTTGGTAACCACGCTTTCACCACCGACGA
ACTGATCTCTCACTTCCAACTGCGTGACGAAGTTCCGTGGTGGAACGTGGTTGGTGACCGTAAATACCAGAAACAGAAACTGG
CGGGTGACCTGGAAACCCTGCGTTCTTACTACCTGGACCGTGGTTACGCGCGTTTCAACATCGACTCTACCCAGGTTTCTCTG
ACCCCGGACAAAAAAGGTATCTACGTTACCGTGAACATCACCGAAGGTGACCAGTACAAACTGTCTGGTGTTGAAGTTTCTGG
TAACCTGGCGGGTCACTCTGCGGAAATCGAACAACTGACCAAAATCGAACCGGGTGAACTGTATAACGGCACCAAAGTTACCA
AAATGGAAGACGACATCAAAAAACTGCTGGGTCGTTACGGTTACGCTTACCCGCGTGTTCAGTCTATGCCGGAAATCAACGAC
GCGGACAAAACCGTTAAACTGCGTGTGAACGTGGACGCGGGTAACCGTTTCTACGTTCGTAAAATCCGTTTTGAAGGTAACGA
CACCTCTAAAGACGCGGTTCTGCGTCGTGAAATGCGTCAGATGGAAGGTGCGTGGCTGGGTTCTGACCTGGTTGACCAGGGTA
AAGAACGTCTGAACCGTCTGGGTTTCTTTGAAACCGTTGACACCGACACCCAGCGTGTTCCGGGTTCCCCGGACCAGGTTGAC
GTTGTTTACAAAGTTAAAGAACGTAACACC**GGATCC**CGTGCCGGCGGGTATGTGGGGCTGGACAATCAGGGCTCGCAGTCTAC
CGGACGGAGCCGCGTCTTGGGTGGCGCGTATGTTAATAGCCTGCTCGGCACAGGTGATCAGCTGCGTATCGATGGTGCTGTGG
GCTACGAGCATGGCGGGCTGGTGAACGGCCGCCTGGATTACAGCATGCTTGTGTCCGGTTATGGCACCCGCGAGGGGGTGGCA
TACAGCCGTCTGGACTACCAGTATGACTTTATGCGGGAGCGCTTCCTGGGGTATTCCGACGACTGGGAGCTCTATGTAAGCCA
TCCGCTCGTCCGCACCGGCACGGCACAGGTGAACCTCCGGGCCTCGGTCGGTCAGTCCTTCCTGACCGACAAATACCCGCAGA
AGTTCTCCCTCTCAAGCGGCAGGGAAGGCAAAAAATCGGCCACCACCGGGACACTGGGTGTGGCGGGCAGCATGGCTACGGTG
CCGGGTGGGGTGACCGGAGCGAGCGTGGACCTGACGGTGGGCCGGATGCTCTACCAGGATGACACCTCCCGCTTCTGGAGCGG
CAGCGACGTGCGCGGTACGGACAGCCACTTCTTCACGTTCAACTACCAGTTGCAGCATGACCAGCAGATTTACGGGCCCCTGC
AGGCCTCCGTTCGTCTGAGCGGTCAGGAAACCAGCCGTAACCTGGATGCGTCCCGCAAGTTCCTGCTGGGCGGGCCGTCTGCG
GTACGTGCCTATGACGTGGGTGCCGGTGCGGTTGACCGCGGTGTTGTGGCCACTGCGGAGGTGAAGTCGACCTGGTCTCTGCC
GGCCGGTACCCGGGTCGGGAAAAGTCCGTTTGTCTCGGTCGGGGCATTTTATGACCACGGTAATGGCCAGCAGAACAGGGATA
ATGCCACAAAAACCGGCCTTCGCCTGACGGATAAAAACGAGGTGAATCTGGGAGGCGGCGGTCTGTTCGCCACCGTGGGTGAC
CCGGGGAACTACGCCGCGACGGTCACCTGGGCCCGCGCGCTTCCGGTAAGGACCCGGTCTCCGGTACCCGGGATGACAACCGC
GTCTGGCTGTCAGCCCTGAAAACGTTCTGA**CTCGAG**

B

>BamA$_{Ec}$
MAMKKLLIASLLFSSATVYGASEGFVVKDIHFEGLQRVAVGAALLSMPVRTGDTVNDEDISNTIRALFAT
GNFEDVRVLRDGDTLLVQVKERPTIASITFSGNKSVKDDMLKQNLEASGVRVGESLDRTTIADIEKGLED
FYYSVGKYSASVKAVVTPLPRNRVDLKLVFQEGVSAEIQQINIVGNHAFTTDELISHFQLRDEVPWWNVV
GDRKYQKQKLAGDLETLRSYYLDRGYARFNIDSTQVSLTPDKKGIYVTVNITEGDQYKLSGVEVSGNLAG
HSAEIEQLTKIEPGELYNGTKVTKMEDDIKKLLGRYGYAYPRVQSMPEINDADKTVKLRVNVDAGNRFYV
RKIRFEGNDTSKDAVLRREMRQMEGAWLGSDLVDQGKERLNRLGFFETVDTDTQRVPGSPDQVDVVYKVK
ERNTGSFNFGIGYGTESGVSFQAGVQQDNWLGTGYAVGINGTKNDYQTYAELSVTNPYFTVDGVSLGGRL
FYNDFQADDADLSDYTNKSYGTDVTLGFPINEYNSLRAGLGYVHNSLSNMQPQVAMWRYLYSMGEHPSTS
DQDNSFKTDDFTFNYGWTYNKLDRGYFPTDGSRVNLTGKVTIPGSDNEYYKVTLDTATYVPIDDDHKWVV
LGRTRWGYGDGLGGKEMPFYENFYAGGSSTVRGFQSNTIGPKAVYFPHQASNYDPDYDYECATQDGAKDL
CKSDDAVGGNAMAVASLEFITPTPFISDKYANSVRTSFFWDMGTVWDTNWDSSQYSGYPDYSDPSNIRMS
AGIALQWMSPLGPLVFSYAQPFKKYDGDKAEQFQFNIGKTW*

>BamA$_{St}$
MAMKKLLIASLLFSSATVYGASEGFVVKDIHFEGLQRVAVGAALLSMPVRTGDTVNDEDISNTIRALFAT
GNFEDVRVLRDGNTLLVQVKERPTIASITFSGNKSVKDDMLKQNLEASGVRVGESLDRTTLSDIEKGLED

FYYSVGKYSASVKAVVTPLPRNRVDLKLVFQEGVSAKIQQINIVGNHAFSTEELISHFQLRDEVPWWNVV
GDRKYQKQKLAGDLETLRSYYLDRGYARFNIDSTQVSLTPDKKGIYITVNITEGDQYKLSGVQVSGNLAG
HSAEIENLTKIEPGELYNGTKVTKMEDDIKKLLGRYGYAYPRVQSQPEINDADKTVKLRVNVDAGNRFYV
RKIRFEGNDTSKDSVLRREMRQMEGAWLGSDLVDQGKERLNRLGFFETVDTDTQRVPGSPDQVDVVYKVK
ERNTGSFNFGIGYGTESGVSFQAGVQQDNWLGTGYSVGINGTKNDYQTYSELSVTNPYFTVDGVSLGGRI
FYNDFQADDADLSDYTNKSYGTDVTLGFPINEYNTLRAGLGYVHNKLSNMQPQIAMDRYLESMGQSADTS
SFAADDFTFNYGWTYNKLDRGYFPTDGSRVNLTGKVTIPGSDNEYYKVSLDTATYVPIDNDHKWVVLGRT
RWGYGDGLGGKEMPFYENFYAGGSSTVRGFQSNTIGPKAVYKNGAHTSWDDNDDYEDCTQESGCKSDDAV
GGNAMAVASLEFITPTPFISEKYANSVRTSFFWDMGTVWDTNWDPSSAPSDVPDYSDPGNIRMSAGIALQ
WMSPLGPLVFSYAQPFKKYDGDKAEQFQFNIGKTW*

>BamA$_{Pa}$
MAMKKLLIASLLFSSATVYGASESFTVSDIRVNGLQRVSAGSVFAALPLNVGETIDDQALVQATRSLFKT
GFFQDIQLGRDGNVLVVTVVERPSISSIEIEGNKAISKEDLLKGLKQSGLAEGEIFQRATLEGVRNELQR
QYVAQGRYSAEINAEVIPQPRNRVALKININEGTVAAISHINVVGNTVFSEEDLTDLFELKTTNWLSFFK
NDDKYAREKLSGDLERLRSYYLDRGYINMDIASTQVSITPDKKHVYITVNINEGEKYTIRDVKLTGDLKV
PEEEVKRLLLVQKGQVFSRKVMTTTSDLITRRLGNEGYTFANVNGVPEAHDDDKTVSVTFVVDPGKRAYV
NRINFRGNTKTEDEVLRREMRQMEGGWASTYLIDQSKARLERLGYFKEVNVETPAVPGTDDQVDVNYSVE
EQPSGSITASVGFAQSAGLILGGSISQNNFLGTGNKVSIGLTRSEYQTRYNFGFVDPYWTVDGVSLGYNA
FYRKTDYDELDVDVASYSVNSLGAGMSIGYPISETSRLTYGLSVQRDQIDTGRYTVDEIYDFLDKEGDNF
TNFKASIGWSESTLNKGVLATRGHSQSLTLETTLPGSDLSFYKIDYRGQVFAPLTDNYTMRFHTELGYGD
GYGSTERLPFYENYYAGGFNSVRGFKDSTLGPRSTPSVARNPDGTPMKNQGPDSKGRYTDPDQDPEAFGG
NILITGGAELLFPLPFVKDQRQLRTVLFWDVGSTFDTDCPTKTTTNCDGIKTDNLASSVGVGLTWITALG
PLSFSLATPIKKPDNAETQVFQFSLGQTF*

>BamA$_{Pm}$
MAMKKLLIASLLFSSATVYGASPFVVKDIRVDGVQAGTEGSVLATLPVRVGQRATDNDIANVVRKLFLSG
QYDDVKASREGNTLVVTVMPKPVISNVVIVGNKSIPDEAIKQNLDANGFKVGDVLNRAKLEEFRKGIVEH
YNSVGRYNAKVDAIVNTLPNNSAEIKIQINEDDVALFKEITFEGNEAFSSGKLADQMELQTDSWWKLFGN
KFDQTQFNKDLETLRSYYLDRGYAQFQILDTDVKLSDDKKEPCLISEEGDLYTVKTRVSGGMWGGMSAEL
APILETIQLNGLFRRTSVLEVEQRNKSKLGERGYATAQVNVHPTFDEQDKTISLDFIVEAGKSYTVRQIR
FEGNTSSADSTLRQEMRQQEGAWLSSELVELGKLRLDRTGYFESVETKTEAIPGSDQVDVIYKVKERNTG
SINFGIGYGTESGLSYQASIKQDNFLGMGSSISLGGTRNDYGTTVNLGYNEPYFTKDGVSLGGNVSFEEY
DSSKSNTSAGYGRTSYGGNLTLGFPVNENNSYYLGVGYTYNKLKNIAPEYNRDLYRQSMKYNDSWTFKSH
DFDLSFGWNYNSLNRGYFPTKGVRANIGGRVTIPGSDNKYYKLNAEAQGFYPLDREHGWVLSSRISASFA
DGFSGKRLPFYQYYSAGGIGSLRGFAYGAIGPNAIYRTRQCPDSYCLVSSDVIGGNAMVTASTELIVPTP
FVADKNQNSVRTSLFVDAASVWNTRWKAEDKAKFAKLNVPDYSDPSRVRASAGVALQWQSPIGPLVFSYA
KPLKKYQGDEIEQFQFSIGGTF*

>BamA$_{At}$
MAMKKLLIASLLFSSATVYGASVISKIDVRGAERSGADSVRSNITIAPGKNFSNSDIDESVKRLYATGYF
SNVSMRVSGSTLVVTVNENQLVNQVVFNGNRKIKDDKLAGIVQTQPMGPFNQAIVTADIARIKEAYSAIG
RSDVEITTQTVSVGQGRVNIAFVINEGERTKIGRIDFIGNNSYSDGRLAAVINTKKSNMLSFLTRKDVYN
EDKLRADEEALRQFYYNRGYADFRVVSSDAVLDESKNEYTISITVDEGKKYDFGNVAVESTVPGVDGSEL
QGLVETRQGASYSAKEVQQSMEAISKRVAGEGYPFARVTPRGDRDMSGNTIGVTYIVDQGERAYVERIEI
RGNTRTRDYVIRREFDISEGDAFNQTIITAAKRRLEALGYFSKVNISTAGGSAPDRVVIVVDVEDQSTGS
FGIGAGYSQNDGVLLEASVEEKNFLGRGQYIRVAAGAGEDDARTYSLSFTEPYFLGYRLAAGFDLFKNQS
KSEDYYNYDEQGFALRVTAPITENLSTTFKYTYKQINYEGKGDWQNNANLAEPYQALIRGEDWTQSILSN
TLNYNTLDDRNMPREGWQAALTNEFAGLGGDSEYYKIYAKARYYYTLSDEYDVIGSLTGQAGHVMPTGDN
LLVFDQFKFGGRQVRGFKNDGIGPRIGSDSIGGTTYFAASAEVTAPMPGVPEDFGLRLAGFVDAGTMYGN
KVSTSQTVKDDNSIRASAGIGVMWASPFGPIRVDYAIPIAKEDYDEEQRFRFGMSNTF*

>BamA$_{Nm}$
MAMKKLLIASLLFSSATVYGASDFTIQDIRVEGLQRTEPSTVFNYLPVKVGDTYNDTHGSAIIKSLYATG
FFDDVRVETADGQLLLTVIERPTIGSLNITGAKMLQNDAIKKNLESFGLAQSQYFNQATLNQAVAGLKEE
YLGRGKLNIQITPKVTKLARNRVDIDITIDEGKSAKITDIEFEGNQVYSDRKLMRQMSLTEGGIWTWLTR
SNQFNEQKFAQDMEKVTDFYQNNGYFDFRILDTDIQTNEDKTKQTIKITVHEGGRFRWGKVSIEGDTNEV
PKAELEKLLTMKPGKWYERQQMTAVLGEIQNRMGSAGYAYSEISVQPLPNAETKTVDFVLHIEPGRKIYV
NEIHITGNNKTRDEVVRRELRQMESAPYDTSKLQRSKERVELLGYFDNVQFDAVPLAGTPDKVDLNMSLT
ERSTGSLDLSAGWVQDTGLVMSAGVSQDNLFGTGKSAALRASRSKTTLNGSLSFTDPYFTADGVSLGYDV
YGKAFDPRKASTSIKQYKTTTAGAGIRMSVPVTEYDRVNFGLVAEHLTVNTYNKAPKHYADFIKKYGKTD
GTDGSFKGWLYKGTVGWGRNKTDSALWPTRGYLTGVNAEIALPGSKLQYYSATHNQTWFFPLSKTFTLML
GGEVGIAGGYGRTKEIPFFENFYGGGLGSVRGYESGTLGPKVYDEYGEKISYGGNKKANVSAELLFPMPG
AKDARTVRLSLFADAGSVWDGKTYDDNSSSATGGRVQNIYGAGNTHKSTFTNELRYSAGGAVTWLSPLGP

MKFSYAYPLKKKPEDEIQRFQFQLGTTF*

>BamA$_{Hp}$
MAMKKLLIASLLFSSATVYGASLENDGSKPNDLTSPKEASQESQKNEAPKNEVQRNEAQKETPQSNQTPK
EMKVKSISYVGLSYMSDMLANEIVKIRVGDIVDSKKIDTAVLALFNQGYFKDVYATFEGGILEFHFDEKA
RIAGVEIKGYGTEKEKDGLKSQMGIKKGDTFDEQKLEHAKTALKTALEGQGYYGSVVEVRTEKVSEGALL
IVFDVNRGDSIYIKQSIYEGSAKLKRRMIESLSANKQRDFMGWMWGLNDGKLRLDQLEYDSMRIQDVYMR
RGYLDAHISSPFLKTDFSTHDAKLHYKVKEGIQYRISDILIEIDNPVVPLKTLEKALKVKRKDVFNIEHL
RADAQILKTEIADKGYAFAVVKPDLDKDEKNGLVKVIYRIEVGDMVYINDVIISGNQRTSDRIIRRELLL
GPKDKYNLTKLRNSENSLRRLGFFSKVKIEEKRVNSSLMDLLVSVEEGRTGSLQFGLGYGSYGGLMLNGS
VSERNLFGTGQSMSLYANIATGGGRSYPGMPKGAGRMFAGNLSLTNPRIFDSWYSSTINLYADYRISYQY
IQQGGGFGVNVGRMLGNRTHVSLGYNLNVTKLLGFSSPLYNRYYSSVNEVVSPRQCSTPASVIINRLSGG
KTPLQPESCSSPGAITTSPEIRGIWDRDYHTPITSSFTLDVSYDNTDDYYFPRNGVIFSSYATMSGLPSS
GTLNSWNGLGGNVRNTKVYGKFAAYHHLQKYLLIDLIARFKTQGGYIFRYNTDDYLPLNSTFYMGGVTTV
RGFRNGSVTPKDEFGLWLGGDGIFTASTELSYGVLKAAKMRLAWFFDFGFLTFKTPTRGSFFYNAPVTTA
NFKDYGVIGAGFERATWRASTGLQIEWISPMGPLVLIFPIAFFNQWGDGNGKKCKGLCFNPNMDDYTQHF
EFSMGTRF*

>BamA$_{Hi}$
MAMKKLLIASLLFSSATVYGASPFVAKDIRVDGVQGDLEQQIRASLPVRAGQRVTDNDVANIVRSLFVSG
RFDDVKAHQEGDVLVVSVVAKSIISDVKIKGNSIIPTEALKQNLDANGFKVGDVLIREKLNEFAKSVKEH
YASVGRYNATVEPIVNTLPNNRAEILIQINEDDKAKLASLTFKGNESVSSSTLQEQMELQPDSWWKLWGN
KFEGAQFEKDLQSIRDYYLNNGYAKAQITKTDVQLNDEKTKVNVTIDVNEGLQYDLRSARIIGNLGGMSA
ELEPLLSALHLNDTFRRSDIADVENAIKAKLGERGYGSATVNSVPDFDDANKTLAITLVVDAGRRLTVRQ
LRFEGNTVSADSTLRQEMRQQEGTWYNSQLVELGKIRLDRTGFFETVENRIDPINGSNDEVDVVYKVKER
NTGSINFGIGYGTESGISYQASVKQDNFLGTGAAVSIAGTKNDYGTSVNLGYTEPYFTKDGVSLGGNVFF
ENYDNSKSDTSSNYKRTTYGSNVTLGFPVNENNSYYVGLGHTYNKISNFALEYNRNLYIQSMKFKGNGIK
TNDFDFSFGWNYNSLNRGYFPTKGVKASLGGRVTIPGSDNKYYKLSADVQGFYPLDRDHLWVVSAKASAG
YANGFGNKRLPFYQTYTAGGIGSLRGFAYGSIGPNAIYAEHGNGNGTFKKISSDVIGGNAITTASAELIV
PTPFVSDKSQNTVRTSLFVDAASVWNTKWKSDKSGLDNNVLKSLPDYGKSSRIRASTGVGFQWQSPIGPL
VFSYAKPIKKYENDDVEQFQFSIGGSF*

>BamA$_{EtpB}$
MAMKKLLIASLLFSSATVYGASEGFVVKDIHFEGLQRVAVGAALLSMPVRTGDTVNDEDISNTIRALFAT
GNFEDVRVLRDGDTLLVQVKERPTIASITFSGNKSVKDDMLKQNLEASGVRVGESLDRTTIADIEKGLED
FYYSVGKYSASVKAVVTPLPRNRVDLKLVFQEGVSAEIQQINIVGNHAFTTDELISHFQLRDEVPWWNVV
GDRKYQKQKLAGDLETLRSYYLDRGYARFNIDSTQVSLTPDKKGIYVTVNITEGDQYKLSGVEVSGNLAG
HSAEIEQLTKIEPGELYNGTKVTKMEDDIKKLLGRYGYAYPRVQSMPEINDADKTVKLRVNVDAGNRFYV
RKIRFEGNDTSKDAVLRREMRQMEGAWLGSDLVDQGKERLNRLGFFETVDTDTQRVPGSPDQVDVVYKVK
ERNT**GS**RAGGYVGLDNQGSQSTGRSRVLGGAYVNSLLGTGDQLRIDGAVGYEHGGLVNGRLDYSMLVSGY
GTREGVAYSRLDYQYDFMRERFLGYSDDWELYVSHPLVRTGTAQVNLRASVGQSFLTDKYPQKFSLSSGR
EGKKSATTGTLGVAGSMATVPGGVTGASVDLTVGRMLYQDDTSRFWSGSDVRGTDSHFFTFNYQLQHDQQ
IYGPLQASVRLSGQETSRNLDASRKFLLGGPSAVRAYDVGAGAVDRGVVATAEVKSTWSLPAGTRVGKSP
FVSVGAFYDHGNGQQNRDNATKTGLRLTDKNEVNLGGGGLFATVGDPGNYAATVTWARASSGKDPVSGTRD
DNRVWLSALKTF*

Fig. S2

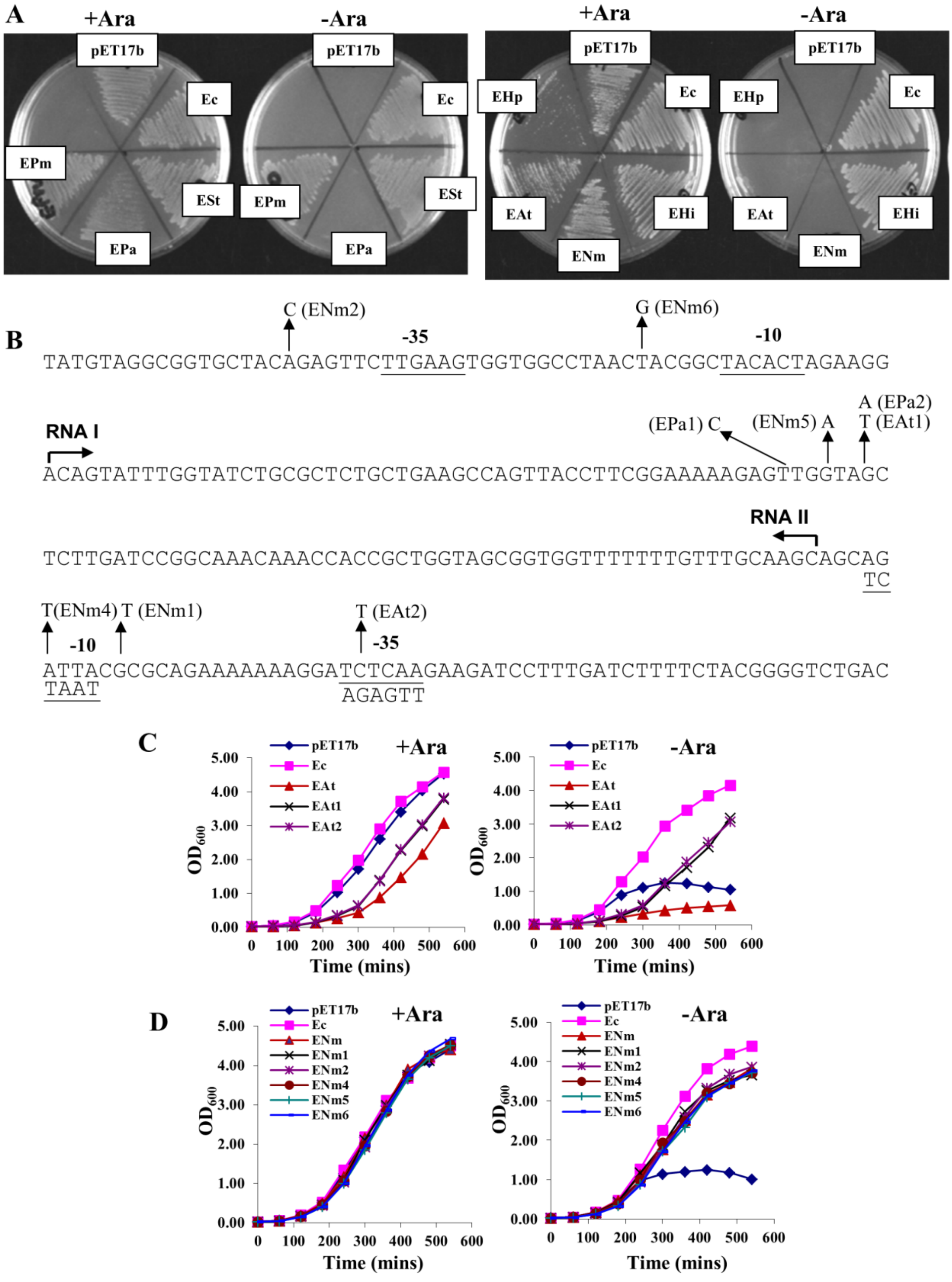
A
+Ara
-Ara
pET17b
Ec
EPm
ESt
EPa
EHp
EAt
EHi
ENm
B
C (ENm2)
G (ENm6)
-35
-10
TATGTAGGCGGTGCTACAGAGTTCTTGAAGTGGTGGCCTAACTACGGCTACACTAGAAGG
RNA I
(EPa1) C
(ENm5) A
A (EPa2)
T (EAt1)
ACAGTATTTGGTATCTGCGCTCTGCTGAAGCCAGTTACCTTCGGAAAAAGAGTTGGTAGC
RNA II
TCTTGATCCGGCAAACAAACCACCGCTGGTAGCGGTGGTTTTTTTGTTTGCAAGCAGCAG
TC
T(ENm4) T (ENm1)
T (EAt2)
-10
-35
ATTACGCGCAGAAAAAAAGGATCTCAAGAAGATCCTTTGATCTTTTCTACGGGGTCTGAC
TAAT
AGAGTT
C
pET17b
Ec
EAt
EAt1
EAt2
+Ara
-Ara
OD600
Time (mins)
D
pET17b
Ec
ENm
ENm1
ENm2
ENm4
ENm5
ENm6
+Ara
-Ara
OD600
Time (mins)

Fig. S3

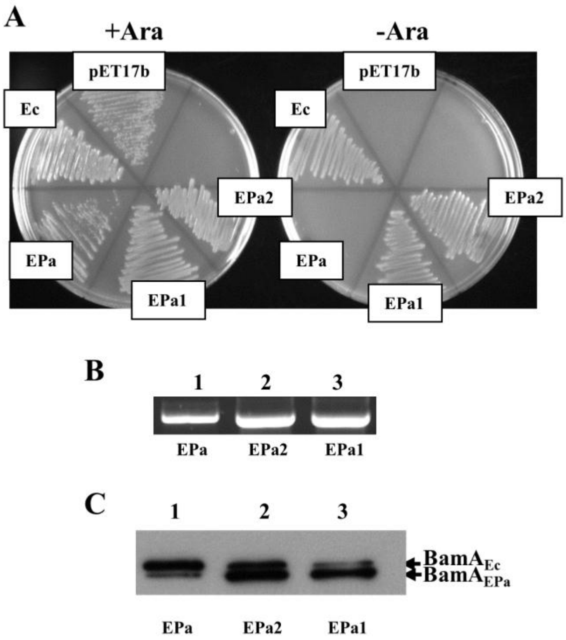
A
+Ara
-Ara
pET17b
Ec
EPa2
EPa
EPa1
B
1
2
3
EPa
EPa2
EPa1
C
1
2
3
BamA$_{Ec}$
BamA$_{EPa}$
EPa
EPa2
EPa1

Fig. S4

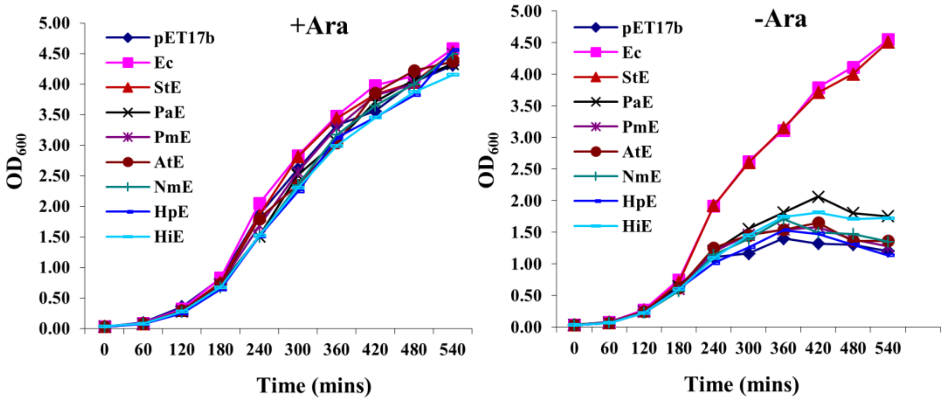
+Ara
-Ara
pET17b
Ec
StE
PaE
PmE
AtE
NmE
HpE
HiE
OD600
Time (mins)
0.00
0.50
1.00
1.50
2.00
2.50
3.00
3.50
4.00
4.50
5.00
0
60
120
180
240
300
360
420
480
540

Fig. S5

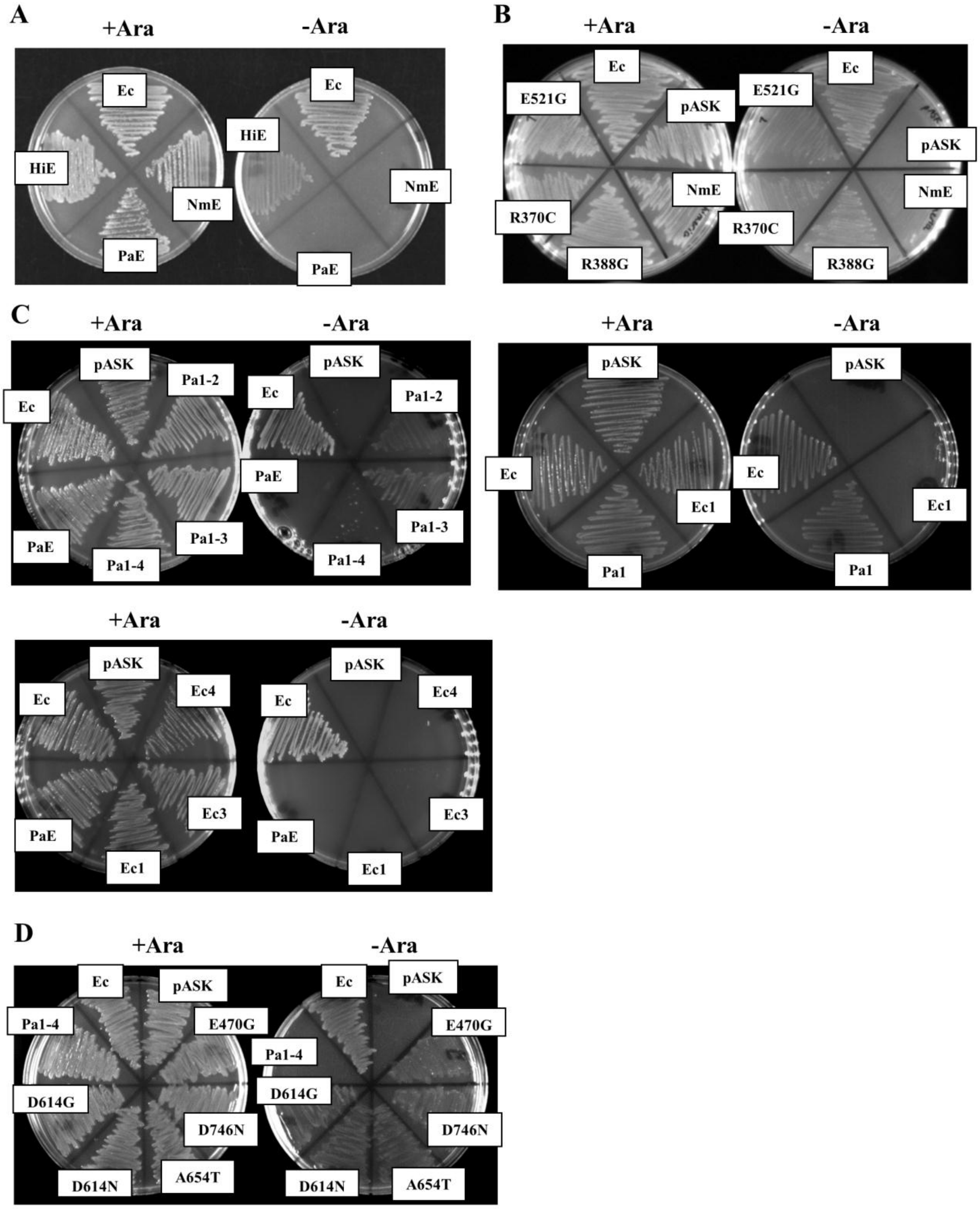
A
+Ara
-Ara
Ec
HiE
NmE
PaE
Ec
HiE
NmE
PaE
B
+Ara
-Ara
Ec
E521G
pASK
NmE
R370C
R388G
E521G
Ec
pASK
NmE
R370C
R388G
C
+Ara
-Ara
pASK
Pa1-2
Ec
PaE
Pa1-3
Pa1-4
Ec
pASK
Pa1-2
PaE
Pa1-3
Pa1-4
+Ara
-Ara
pASK
Ec
Ec1
Pa1
pASK
Ec
Ec1
Pa1
+Ara
-Ara
pASK
Ec
Ec4
PaE
Ec3
Ec1
pASK
Ec
Ec4
PaE
Ec3
Ec1
D
+Ara
-Ara
Ec
pASK
Pa1-4
E470G
D614G
D746N
D614N
A654T
Ec
pASK
E470G
Pa1-4
D614G
D746N
D614N
A654T

Fig. S6

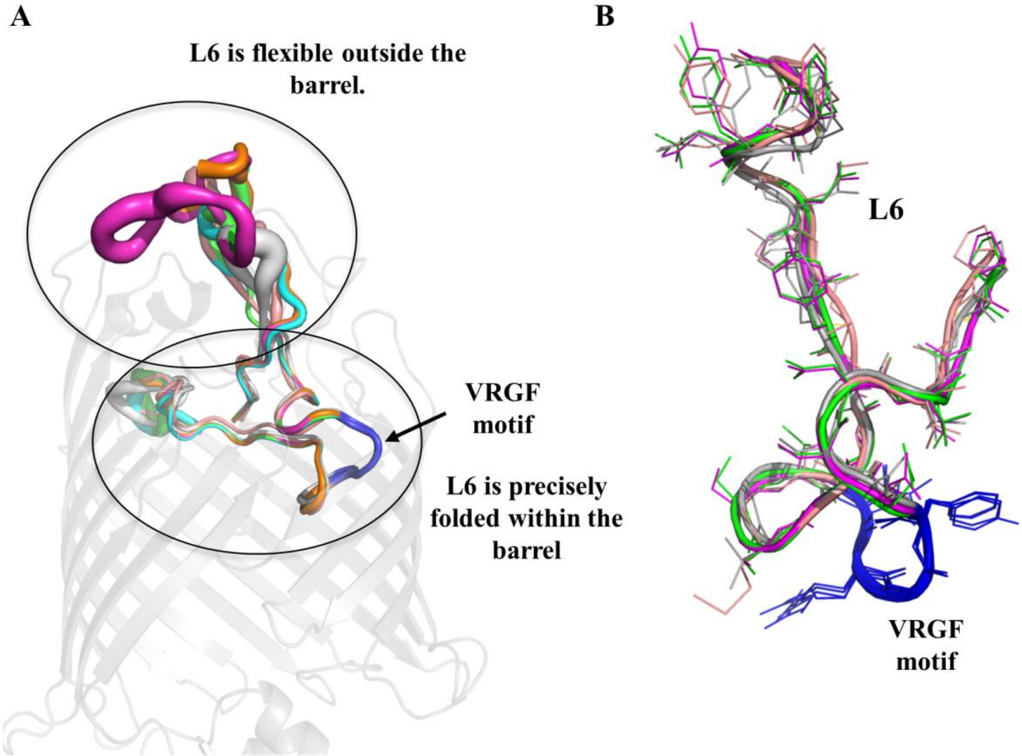

A
L6 is flexible outside the barrel.
VRGF motif
L6 is precisely folded within the barrel
B
L6
VRGF motif
